# Ethnobotany of the medicinal plants used by the ethnic communities of Kerman province, Southeast Iran

**DOI:** 10.1186/s13002-021-00438-z

**Published:** 2021-04-28

**Authors:** Seyed Hamzeh Hosseini, Hossein Bibak, Abdollah Ramzani Ghara, Amirhossein Sahebkar, Abolfaz Shakeri

**Affiliations:** 1 Department of Biology, Faculty of Science, University of Jiroft, Jiroft, Iran; 2 Biotechnology Research Center, Pharmaceutical Technology Institute, Mashhad University of Medical Sciences, Mashhad, Iran; 3 Neurogenic Inflammation Research Center, Mashhad University of Medical Sciences, Mashhad, Iran; 4 Department of Pharmacognosy, School of Pharmacy, Mashhad University of Medical Sciences, Mashhad, Iran

**Keywords:** Ethnobotany, Medicinal plants, ICPC category, Kerman province, Iran

## Abstract

**Background:**

Traditional medicine is a major component in the primary healthcare system in the southeast of Iran, which has a rich floral diversity. However, there is no comprehensive report on the use of medicinal herbs in this specific region. This traditional usage of medicinal plants by local communities could serve as a source for pharmacological and phytochemical studies. The main objective of this study was to identify ethnopharmacological knowledge on medicinal plant species and their local healing applications by the folk communities of Kerman province in the southeast of Iran.

**Methods:**

In this cross-sectional study, data were collected from 217 herbal healers using semi-structured questionnaires, open interviews, and field surveys. Factors including use reports (UR) for each species, frequency of citation (FC), and informant consensus factor (ICF) were used to analyze the data. Plant species were identified by botanists through standard taxonomic methods.

**Results:**

A total of 402 medicinal plants were used in healing practices by the local communities of Kerman province. These species belong to 273 genera of 73 families, among which 367 species are dicotyledons, 27 are monocotyledons, 7 species are cryptogam, and one species is gymnosperm. An important implication from the current study is the identification of the traditional medicinal use of 292 plant species in this region for the first time. Asteraceae, Apiaceae, Lamiaceae, and Fabaceae were the dominant medicinally utilized plant families, respectively. Leaf, flower, fruit, and seed were the most common plant parts used. Generally, crude drugs were used in the form of decoction, followed by poultice and infusion forms. Moreover, oral route is considered as the most common administration route followed by topical route. Endocrine (diabetes), dermatological, gastrointestinal, and respiratory problems were ranked as the most frequent ailment categories for which medicinal plants in this region were applied, respectively. Our findings suggested dominant use of Asteraceae and Apiaceae plants for the treatment of gastrointestinal disorders, Lamiaceae plants for respiratory and gastrointestinal ailments, and Apocynaceae plants for dermatological problems.

**Conclusion:**

Our findings suggested that Asteraceae and Apiaceae plants were used for the treatment of gastrointestinal disorders, Lamiaceae plants for respiratory and gastrointestinal ailments, and Apocynaceae and Euphorbiaceae plants for dermatological problems. Among the medicinal plants with high UR and new ethnobotanical uses, *Rhazya stricta* was used for wound healing, *Calotropis procera*, *Clematis ispahanica* and *Euphorbia* spp. for eczema, *Cionura erecta* for the treatment of cough, *Launaea acanthodes* for the treatment of gastrointestinal parasites, *Berberis integrrima* as an antidiabetic medicinal herb, *Dracocephalum polychaetum* and *Rydingia persica* for various types of chronic diseases, *Citrus limon* and *Citrus aurantium* for the treatment of ocular diseases and making the traditional kohl, *Calendula officinalis* for the treatment of pterygium and *Prosopis farcta* for preventing nasal bleeding. The identified medicinal plants can be further evaluated for their pharmacological activity and underlying mechanisms of action.

## Introduction

According to the reports, medicinal use of plants dates back to at least 60,000 years. During this time, traditional systems of medicines have employed medicinal plants and their derivatives as valuable sources of new biologically active compounds and have been clinically practiced all over the world [1]. Until now, approximately 80% of the world’s population still use traditional herbal medicines [2]. In fact, herbal medicines can serve as complementary or alternative therapies for different types of diseases because of their low cost, availability, and generally fewer side effects [3]. Several FDA-approved drugs including artemisinin (from *Artemisia annua*), quinine (from *Cinchona officinalis*), vinblastine, vincristine, vinorelbine (from *Vinca rosea*), and etoposide (from Mayapple) primarily originate from traditional herbal medicines [4]. It has been estimated that nearly 400,000 flowering plant species exist on earth, among which only 6% have been evaluated for their biological properties, and still more than 90% remains unexplored [5]. Therefore, ethnobotanical study of medicinal plants provides valuable information for the synthesis of new drugs.

Around 8000 plant species have been listed in Iran, of which 2300 species have medicinal properties among which 75% (1728) are endemic species in Iran [6, 7].

Kerman province with 23 cities and 171,993 square kilometers area has covered about 11% of the land area of Iran [8], located in the southeast of this country, and bordered by 5 provinces of Yazd, South Khorasan, Hormozgan, Fars, and Sistan and Baluchestan. It has a unique biodiversity due to its diverse natural resources and climatic conditions including desert and semi-desert in the north, and dry, mountainous and Mediterranean in the south. Kerman province is a vast plain with the lowest altitude in Lut desert (300 m) and the highest altitude in the mountaintop of Hezar (4419 m) [8]. Based on the traditional pharmacopoeia and medicinal plant reports in some parts of this province, medicinal herbs mostly belong to the families of Labiatae, Rosaceae, Papilionacae, Compositae, and Umbelliferae, and the genera of *Salvia*, *Nepeta*, *Artemisia*, *Astragalus*, *Ferula*, *Plantago*, *Ephedra*, and *Amygdalus* [9, 10]*.*

From the cultural point of view, Kerman province has around 89 tribal communities (including Baluch, Turkish, and Fars), most of them still being partially dependent on the medicinal plants. Therefore, this province is home to different cultures and beliefs resulting in rich traditional knowledge and traditional medicine practices. For example, the old city of Jiroft in the southeastern Kerman province dates to about 5000 years ago, which, according to the reports, is the beginning of human civilization [11]. In this respect, traditional medicine has played a key role in Iranian culture and civilization [12]. Therefore, this rich traditional knowledge is useful not only in the ancient medical systems but also in the present healthcare systems [10], especially for primary health care needs [13]. In fact, the dependence of the folk communities in Kerman on the medicinal herbs is not only due to the low availability to the health care system, but it is also rooted in the Iranian-rich culture of traditional medicine [14, 15]. For example, in the face of epidemiological diseases (e.g., cholera and colds), scientists of the Iranian traditional medicine (ITM) such as Avicenna, Rhazes, and Aghili Alavi Shirazi have suggested prescription of various herbal remedies. At present, the locals of Kerman, based on their ancient knowledge, utilize herbal medicines such as *Thymus fedtschenkoi*, *Zataria multiflora*, *Dracocephalum polychaetum*, and *Glycyrrhiza glabra* in the treatment of epidemics. Generally, Kerman province with a diverse climate and biodiversity is home to various cultures (from the prehistoric times to the present) and the center of agriculture in Iran [10, 16]. Accordingly, in some areas of this region, certain non-registered herbaceous species are used that can be obtained by the local people. There are many villages and nomadic districts that are largely dependent on the ethnomedicinal knowledge for primary health care, with many specific traditional herbal medicine practices in this region that have not been recorded anywhere else. Hence, the current study aimed to carefully investigate and record the ethnobotanical knowledge of the whole districts and cultures, particularly subcultures that had the maximum dependence on the traditional health care system of the Kerman province.

## Materials and methods

### Study area

The present study was carried out in Kerman province in the southeast of Iran with 23 cities and 3,164,716 inhabitants. Regarding population, the most populated city is Kerman with the following other cities as progressively less populated: Jiroft, Sirjan, Rafsanjan, Kahnuj, Rudbar, Anbarabad, Qale Ganj, Manojan, Faryab, Zarand, Bam, Fahraj, Narmashir, Rigan, ShahrBabak, Baft, Rabor, Orzueeyeh, Bardsir, Ravar, Anar, and Koohbanan. In the current study, in each city, skilled herbalists, nomadic districts, and key villages were selected for data sampling. Kerman province is located between the 55 min and 25° to 32° north latitude and 26 min and 53° to 29 min and 59° east of the Greenwich meridian, as the largest province in Iran with the total area of 183.285 km^2^, and the elevation of 400 to 4501 m above the sea level. About 6.3 million hectares of deserts of Iran (equivalent to 20%) are located in Kerman province. The area of the forests of Kerman province is 1.3 million hectares and belongs to the two vegetation regions of Irano-Turanian and Khaleej-Omani.

**Fig. 1:**
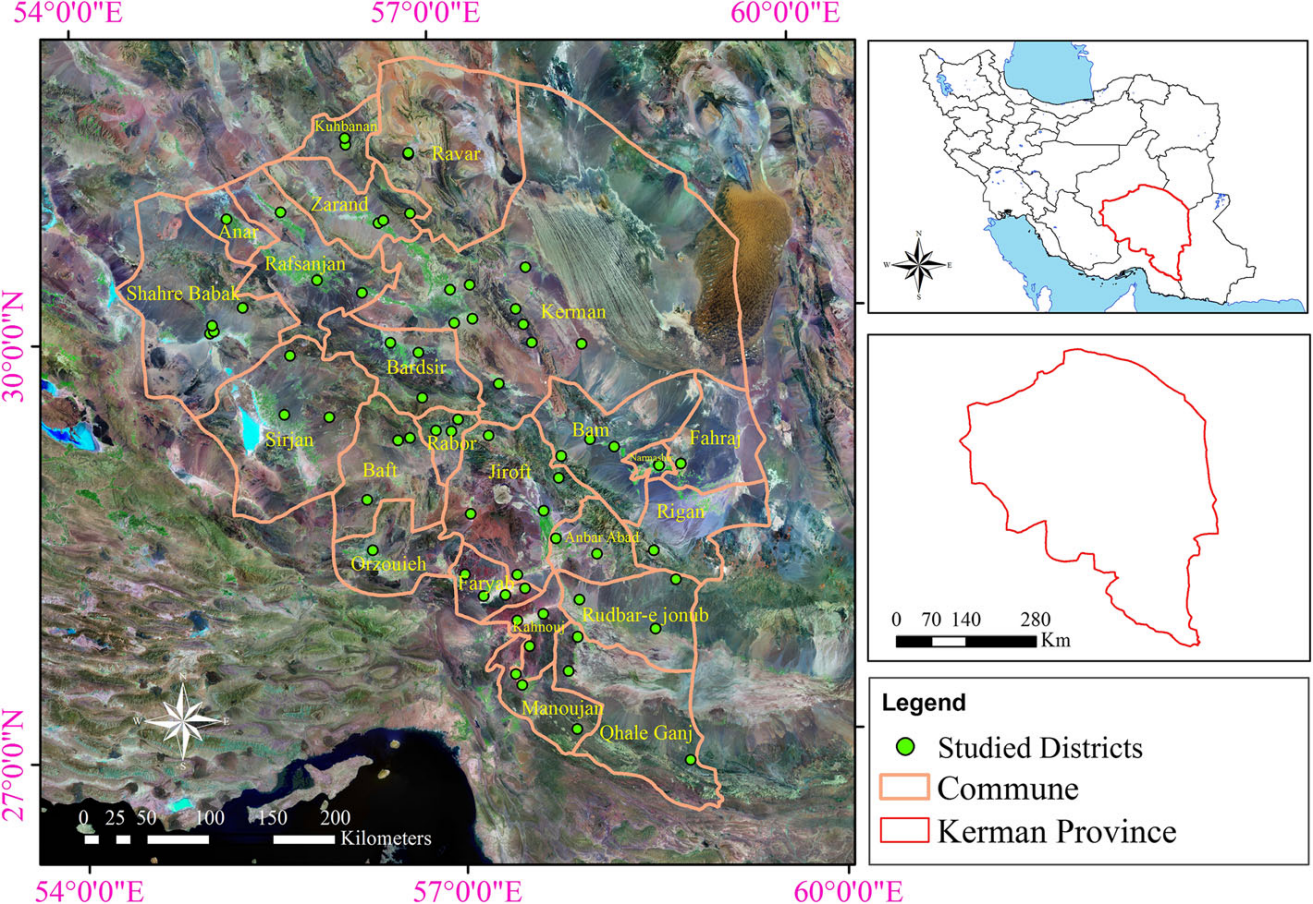
Study area, Kerman province, southeast Iran

Species of the Irano-Turanian forest of Kerman are comprised of *Pistacia atlantica*, *Pistacia khinjuk*, *Juniperus excels*, *Prunus scoparia*, *Crataegus azarolus*, *Celtis australis* in the mountainous area, and *Haloxylon* spp*.* and *Calligonum* spp. in the desert area. Also, the species of Khaleej-Omani include *Calotropis procera*, *Tamarix* spp*.*, *Prosopis* spp., and *Ziziphus* spp*.* and the endemic rare species of *Tecomella undulate* with the local names Golpar and Anar sheytan (Fig. 1).

### Plant identification

Based on the maps, access roads, natural features, vegetation, and subcultures in the study area, each city was classified into districts. Then, the number of the informants was determined. The plant specimens were collected during the field surveys from nomadic, rural, and urban areas of Kerman province from 2017 to 2019. The herbarium specimens were prepared following standard methods [17–19] and identified with the help of Herbarium of the University of Jiroft and Kerman Agricultural and Natural Resources Research and Education Center. Nomenclature was corrected using an online database (the international plant names index and the plant lists). The voucher herbarium specimens were deposited in the herbarium of the Department of Plant Biology, University of Jiroft, Jiroft, Iran. The voucher specimens were identified by one of the authors (H.B) and reaffirmed by taxonomic experts from the Department of Plant Biology at the herbarium of the University of Jiroft.

### Ethnobotanical data collection

After classification of each city into districts, ethnobotanical surveys were carried out from October 2017 to the end of May 2019. The ethnobotanical data was collected using field surveys, open interviews, and semi-structured questionnaires. A total of 217 local informants (91 females and 126 males) aged between 30 and 79 years old were interviewed. Demographic properties including educational level, gender, age group, and occupation are recorded in Table 1. Also, geographical location and altitude (the lowest being 409 m in Manojan and the highest being 2800 m in Lalehzar) of each district are recorded in Table 2. Furthermore, information on local name, medicinal use, part(s) of the plant used, preparation, and administration methods is recorded (Table 3).

**Table 1:** Demographic profile of the local healers (n=217)

Characteristics		Abundance	Relative abundance
Gender	Male	126	57.60
	Female	91	42.60
Education	Primary level	103	47.47
	Secondary level	86	39.63
	Graduate	28	12.90
Age group	30-45	51	23.50
	46-60	132	60.83
	61-79	34	15.67
Occupation	Nomadic tribe	94	43.31
	Farmer	78	35.94
	Herbal healer	45	20.74

**Table 2:** Studied districts in the Kerman province with in-detail demographic characteristics of the local informants

Area	Village- nomadic district	Altitude	Location		Number of informants	Gender	
			Latitude	Longitude		Male	Female
Jiroft	Esfandaghe	1724	515518	3173088	6	5	1
	Boluk	653	550080	3123049	4	3	1
	Northen Jebal barez	1973	586377	3198024	5	4	1
	Sardooiyeh	2622	532724	3234040	13	7	6
	Central part	682	573397	3172706	8	5	3
Anbarabad	Southern Jebal barez	890	613586	3136457	9	7	2
	Central part	597	581969	3150210	5	2	3
Kahnuj	Dehkehan	810	556930	3066254	4	2	2
	Dehzard	518	548076	3086568	2	-	2
	Central part	513	568775	3090872	5	4	1
Faryab	Sargorij	692	555482	3111865	4	2	2
	Mehuiyeh	649	539817	3107390	3	2	1
	Moordan	1118	508786	3125367	1	1	-
	Central part	659	522398	3107510	1	1	-
Rudbar-e Jonub	Zehkalut	385	656761	3075068	2	-	2
	Mil-e Farhad	816	674888	3113175	1	1	-
	Central part	488	597867	3100902	2	1	1
Qaleh Ganj	Sorkhqleh	439	595145	3071601	1	1	-
	Maarz	937	591255	2998867	3	2	1
	Central part	407	586721	3045020	4	1	3
Manujan	Nodej	464	544955	3044682	2	1	1
	Central part	358	549652	3035783	2	1	1
Baft	Bazenjan	2346	470709	3235218	2	-	2
	Khabr	2039	434312	3188268	2	1	1
	Central part	2262	461018	3233719	5	3	2
Rabor	Sardmeshk	2495	509371	3247951	3	1	2
	Qanat Malek	2300	503669	3238948	3	1	2
	Central part	2332	491445	3240172	4	3	1
Sirjan	Balvard	2035	407806	3254993	6	4	2
	Pariz	2313	379188	3305535	4	2	2
	Central part	1744	372254	3258624	5	1	4
Rafsanjan	Bahreman	1330	377582	3419214	3	1	2
	Kabutatkhan	1662	438574	3352462	3	3	-
	Central part	1515	403788	3364209	2	1	1
Anar	Central part	1414	334675	3416022	4	3	1
Ravar	Central part	1181	481234	3459578	4	2	2
Zarand	Hotkan	2325	479943	3412892	3	2	1
	Mahmud abad	1651	454636	3406622	3	1	2
	Central part	1656	458637	3408681	4	3	1
Shahr Babak	Estabraq	1794	316665	3326121	2	2	-
	Mehrabad	1817	319991	3327739	2	1	1
	Meymand	2218	343549	3345546	5	2	3
	Central part	1840	318777	3332404	3	1	2
Koohbanan	Joz	1989	431829	3470143	2	2	-
	Central part	1991	431755	3475352	2	1	1
Bam	Dehbakri	2039	589237	3215060	5	4	1
	Central part	1068	631396	3220002	2	1	1
Fahraj	Central part	678	683329	3204470	3	1	2
Narmashir	Central part	753	665948	3204479	2	2	-
Rigan	Koosha	1586	658487	3137050	2	-	2
	Central part	756	666076	3203853	2	-	2
Orzueeyeh	Central part	1044	436602	3148052	3	2	1
Bardsir	Lalehzar	2800	481843	3266686	4	3	1
	Negar	2090	480752	3302559	3	3	-
	Central part	2042	459122	3311509	2	1	1
Kerman	Mahan	1854	524742	3327108	3	1	2
	Shahdad	429	568791	3365494	3	1	2
	Rayen	2161	542947	3274724	4	2	2
	Jopar	1887	510333	3324762	5	4	1
	Central part	1757	508230	3351251	11	7	4
Total					217	126	91

**Table 3:** Medicinal plants used by ethnic communities in the Kerman province

Family	Scientific name	Local name (Persian); Voucher no.	Plant part used	Medicinal use (UR)	ICPC category	Preparation	Mode of application	(UR)	A, B, C
Acanthaceae	*Blepharis edulis* (Forssk.) Pers.	Anjereh 503	Leaf, Seed	Wound healing (3), Ear ache (2), Eye ache (2), Sore throat (3)	DER-S	Poultice, Decoction	Topical	10	A
Amaranthaceae	*Amaranthus retroflexus* L*.*	Sorkhmaghz 598	Leaf	Jaundice (18)	GAS-D	Decoction	Oral	18	B, C
Amaranthaceae	*Anabasis aphylla* L.	Aldorak 596	Aerial parts	Weight loss (3), Constipation (1)	OTH-A, GAS-D	Aromatic water	Oral	4	B, C
Amaranthaceae	*Seidlitzia rosmarinus* Bunge ex Boiss.	Shoor 594	Aerial parts	Washing powder (5)	-, -	Powder	-	5	B, C
Amaranthaceae	*Salsola incanescens* C.A. Mey.	Jar 593	Aerial parts	Washing powder (2)	-, -	Powder	-	2	B, C
Amaranthaceae	*Dysphania botrys* (L.) Mosyakin & Clemants	Dermeneh 595	Young flowering branches	Beauty of skin and hair (4)	DER-S	Oil	Topical	4	B, C
Amaranthaceae	*Suaeda aegyptiaca* (Hasselq.) Zohary	Somsil 597	Aerial parts	Blood purifier (4), Anemia (29), Vegetable (48)	OTH-A, Blood-B	Vegetable	Oral	81	B, C
Amaryllidaceae	*Allium atroviolaceum* Boiss.	Piaze vahshi 268	Bulb	Reduce blood sugar (2)	MET-T	Vegetable	Oral	2	B, C
Amaryllidaceae	*Allium iranicum* (Wendelbo) Wendelbo.	Serit 245	Leaf	Aromatic (4), Flavoring of food (33), Digestive (6)	NER-N, -, GAS-D	Spice	Oral	43	B, C
Amaryllidaceae	*Ixiolirion tataricum* (Pall.) Schult. & Schult.f.	Kheyaroo 269	Leaf and flower	Pickle (6)	OTH-A	Vegetable	Oral	6	B, C
Amaryllidaceae	*Narcissus tazetta* L.	Narges 267	Bulb, Leaf	Face rash treatment (3), Sterility treatment (2), Gastric discomfort (2), Blood coagulation (4), Anti-depressants (5)	DER-S, DER-S, GAS-D, Blood-B, NER-N	Mask, Decoction, infusion	Topical, Oral	16	A, C
Anacardiaceae	*Pistacia atlantica* Desf*.*	Baneh 623	Seed	Bone and joint pains (5), Burn healing (68), Wound healing (26), Eczema (34)	SKE-L,DER-S, DER-S, DER-S	Oil	Topical	208	A
			Olibanum	Scar (39)	DER-S	Poultice	Topical		
			Olibanum	Lung infections (1)	RES-R	Fume	Inhale		
			Leaf, Olibanum	Stomach ulcers (31), Toothache (2)	GAS-D, GAS-D	Extract, Gum	Oral		
			Fruit, Olibanum	Disinfectant (2)	OTH-A	Edible	Oral		
Anacardiaceae	*Pistacia khinjuk* Stocks	Kasour 621	Seed, Leaf	Hemorrhoid (1), Stomachache (9), Toothache (2), Memory Improvement (2), Jaundice (1)	CAR-K, GAS-D, GAS-D, NER-N, GAS-D	Nuts, Mixed with date, Gum	Oral	15	A
Anacardiaceae	*Pistacia vera* L*.*	Pesteh 620	Fruit	Reinforcing sexual desire (2), Anti-nausea (1), Anti-diarrhea (1), Constipation (3)	OTH-A, GAS-D, GAS-D, GAS-D,	Nuts	Oral	7	A
Apiaceae	*Ammi majus* L.	Golsefid 220	Fruit	Anti-nausea (5), diuretic (4)	GAS-D, URO-U	Decoction	Oral	9	A
Apiaceae	*Anethum graveolens* L.	Maitokhm 234	Seed	Blood fat (3), Gastric discomfort (32), Energetic (2), Reduce blood sugar (2), Joint pain (5)	Blood-B, GAS-D, OTH-A, MET-T, SKE-L	Decoction	Oral	44	A
Apiaceae	*Apium graveolens* L*.*	Karafs 221	Aerial parts	Relaxing (1), Disinfectant (2), Flavoring of food (40)	NER-N, OTH-A, -	Mixed with food, Vegetable	Oral	43	B, C
Apiaceae	*Bunium persicum* (Boiss.) B.Fedtsch.	Ziresiah 227	Seed	Menstrual disorders (30), Flavoring of food (115), Digestive (2), Parasite repellent (3), Carminative (4), Antispasmodic (31),	GYN-X, OTH-A, GAS-D, GAS-D, GAS-D, NER-N	Decoction, Infusion, Mixed with food	Oral	185	A
Apiaceae	*Carum carvi* L.	Ziresiah 230	Fruit	Carminative (6), Relaxing (1)	GAS-D, NER-N	Infusion	Oral	7	A, C
Apiaceae	*Conium maculatum* L.	Showkaran 233	Whole plant	Cough (4), Respiratory ailments (6)	RES-R, RES-R	Decoction	Oral	10	A
Apiaceae	*Coriandrum sativum* L.	Geshniz 238	Leaf, Seed, Aerial parts	Reduce blood sugar (3), Intestinal infections (2), Blood fat (4), lactiferous (5), Flavoring of food (18) Carminative (3)	MET-T, GAS-D, Blood-B, PRE-W, OTH-A, GAS-D	Decoction, Dried vegetable	Oral	35	A
Apiaceae	*Cuminum cyminum* L.	Ziresabz 225	Seed	Menstrual disorders (10), Flavoring of food (14), Body tonic (5)	GYN-X, -, OTH-A	Decoction, Infusion, Mixed with food	Oral	29	A
Apiaceae	*Daucus carota* L.	Havij 235	Bulb	Anemia (8), Sight enhancement (7), Appetizing (3)	Blood-B, EYE-F, OTH-A	Salad	oral	18	A, C
Apiaceae	*Dorema ammoniacum* D. Don	Oshtork 258	Gum	Disinfectant (4), Edible (7), Infectious wound healing (11)	OTH-A, OTH-A, DER-S	Powder, Mask, Poultice, Vegetable	Oral	22	A
Apiaceae	*Dorema aucheri* Boiss.	Oshtork 249	Gum	Disinfectant (4), Edible (7), Infectious wound (11)	OTH-A, OTH-A, DER-S	Powder, Mask, Poultice, Vegetable	Oral	22	A
Apiaceae	*Ducrosia anethifolia* (DC.) Boiss.	Reshkak 291	Leaf, Seed	Abdominal pains (6), Body tonic (7), Child Carminative (9)	GAS-D, OTH-A, GAS-D	Infusion	Oral	22	A
Apiaceae	*Ducrosia assadii* Alava.	Reshkak 237	Leaf and fruit	Wound and burns healing (8)	DER-S	Oil	Topical	8	A
Apiaceae	*Eryngium billardieri* Delile	Chichagh 240	Aerial parts	Expectorant (4), Bronchitis (4), Antispasmodic (1), Carminative (2), Cough (4), Pain relief (3), Reduce rheumatic pain (1)	RES-R, RES-R, NER-N, GAS-D, RES-R, SKE-L	Decoction	Oral	19	B, C
Apiaceae	*Eryngium bungei* Boiss.	Shoochagh 242	Aerial parts	Pain relief (4)	NER-N	Decoction	Oral	4	B
Apiaceae	*Ferula assa-foetida* L.	Anghouze 245	Gum	Parasite intestine (16), Expectorant (2), Menstrual disorders (2), Gastritis (5)	GAS-D, RES-R, GYN-X, GAS-D	Fume, Infusion	Oral	25	A
Apiaceae	*Ferula gummosa* Boiss*.*	Anghouzeh 236	Gum	Cough (6), Laxative (3)	RES-R, GAS-D	Infusion	Oral	9	A
Apiaceae	*Ferula oopoda* (Boiss. & Buhse) Boiss.	Anghouzeh 252	Latex, Fruit	Toothache (35), Carminative (18), Intestinal parasite (30), Tooth infection (10), Expectorant (4)	GAS-D, GAS-D, GAS-D, GAS-D, RES-R	Poultice, Decoction	Oral	97	A
Apiaceae	*Ferula ovina* (Boiss.) Boiss.	Anghouze shirin 247	Aerial parts	Carminative (15)	GAS-D	Decoction	Oral	15	A
Apiaceae	*Ferula persica* Willd.	Anghouzeh 250	Latex, Fruit	Cough (6), Laxative (3)	RES-R, GAS-D	Decoction	Oral	9	A
Apiaceae	*Ferula szowitziana* DC.	Anghouze 254	Latex	Stomachache (6)	GAS-D	Cooked with meat and vegetables	Oral	6	A
Apiaceae	*Ferulago angulata* (Schltdl.) Boiss.	Garchi 257	Aerial parts	Carminative (6), Flavoring of butter (1), Aromatherapy (1)	GAS-D, OTH-A	Decoction, Powder, Dressing	Oral	8	A
Apiaceae	*Foeniculum vulgare* Mill*.*	Badiuon 244	Seed	Carminative (41), Gastric discomfort (27), Bone and joint pains (12), Asthma (1), Digestive (11)	GAS-D, GAS-D, SKE-L, RES-R, GAS-D	Decoction	Oral	92	A
Apiaceae	*Pulicaria undulata* (L.) C.A.Mey.	Sooteh 259	Leaf	Dysentery (10), Wound healing (3)	GAS-D, DER-S	Decoction, Poultice	Oral, Topical	14	A, C
Apiaceae	*Heracleum persicum* Desf. ex Fisch., C.A.Mey. & Avé-Lall.	Golpar 258	Fruit, Flower	Relaxing (8)	NER-N	Aromatic water	Oral	8	B, C
Apiaceae	*Levisticum officinale* W.D.J.Koch	Karasm 262	Aerial part	Carminative (5), Gastric discomfort (10), Blood pressure (19)	GAS-D, GAS-D, CAR-K	Aromatic water	Oral	34	B, C
Apiaceae	*Levisticum persicum* Freyn & Bornm.	Karasm 260	Aerial parts, Stem	Pickle (3), Aromatherapy (1), Flavoring of food (2)	OTH-A, OTH-A	Decoction, Dressing, Powder	Oral	5	A
Apiaceae	*Petroselinum crispum* (Mill.) Fuss	Jafari 229	Young branches	Urinary stone (3), Digestive (12), Anemia (5)	URO-U, GAS-D, Blood-B	Decoction	Oral	20	A
Apiaceae	*Pimpinella saxifraga* L*.*	Jafari Kouhi 263	Leaf	Stomachache (21)	GAS-D	Decoction	Oral	21	A
Apiaceae	*Platychaete aucheri* (Boiss.) Boiss.	Zarbarook 255	Leaf	Asthma (7)	RES-R	Decoction	Oral	7	A, C
Apiaceae	*Prangos cheilanthifolia* Boiss.	Sekbinch 280	Young branches, Gum	Carminative (5)	GAS-D	Mixed with food	Oral	5	B, C
Apiaceae	*Prangos ferulacea* (L.) Lindl.	Garchi 253	Foliage	Flavoring of dairy (8), Parasite repellent (6), Toothache (7), Carminative (3), Acne (2), Infectious wound (5)	-, GAS-D, GAS-D, GAS-D, DER-S, DER-S	Edible, Decoction, Poultice	Oral, Topical	41	B, C
Apiaceae	*Prangos uloptera* DC*.*	Jashir 270	Young branches	Carminative (15), Body tonic (17)	GAS-D, OTH-A	Mixed with food	Oral	32	A, C
Apiaceae	*Psammogeton stocksii* (Boiss.) Nasir	Izbok 273	Aerial parts	Febrifuge (4)	OTH-A	Decoction	Oral	4	B, C
Apiaceae	*Scandix stellata* Banks & Sol.	Badiyan koohi 275	Whole plant	Body tonic (25), Stomach tonic (13)	OTH-A, GAS-D	Decoction	Oral	38	B, C
Apiaceae	*Trachyspermum ammi* (L.) Sprague	Kaserk 277	Fruit	Stomachache (19), Carminative (7)	GAS-D, GAS-D	Decoction	Oral	26	A
Apiaceae	*Pycnocycla spinosa* Decne*.*	Sagdandan 279	Root	Scorpion bite (1)	OTH-A	Decoction	Oral	2	B, C
Apiaceae	*Pycnocycla bashgardiana* Mozaff*.*	Pvander 288	Flower	Stomachache (2)	GAS-D	Decoction	Oral	3	B, C
Apocynaceae	*Calotropis procera* (Aiton) Dryand.	Kark 849	Latex	Eczema (98), Scorpion bite (9), Earache (4), Toothache (6), Cancer (1)	DER-S, DER-S, Ear-H, GAS-D, CAN-C	Latex	Topical, Oral	151	B
			Leaf	Reduce blood sugar (11), Bruise (10)	MET-T, SKE-L	Poultice	Topical		
Apocynaceae	*Cionura erecta* (L.) Griseb.	Jaze sabz 852	Leaf	Sore throat (3), Expectorant (77), Cough (87)	OTH-A, RES-R, RES-R	Decoction	Oral	167	B, C
Apocynaceae	*Nerium oleander* L.	Gish 861	Leaf	Stomachache (3), Skin diseases (1)	GAS-D	Decoction, Latex	Oral	4	A, C
Apocynaceae	*Pergularia tomentosa* L.	Keshtook 855	Latex	Eczema (2), Constipation (1), Parasite repellent (2), Cancer (1)	DER-S, GAS-D, CAN-C	Poultice, Decoction	Topical, Oral	15	B, C
			Whole parts	Hair removal of animal skin (9)	OTH-A	Decoction	Topical		
Apocynaceae	*Periploca aphylla* Decne.	Shirbadam 853	Latex, Aerial parts	Burn healing (3), Skin inflammation (5)	DER-S, DER-S	Mask, Poultice	Topical	8	B, C
Apocynaceae	*Rhazya stricta* Decne.	Eshbarg 859	Leaf	Scorpion and snake bite (5), Sore throat (2), Febrifuge (41), Ear ache (1), Eye ache (1)	DER-S	Decoction	Bath	173	B
			Leaf	Wound healing (63), Joint pains (57), Reduce blood sugar (3)	DER-S, SKE-L, MET-T	Poultice	Topical		
Arecaceae	*Phoenix dactylifera* L.	Mogh 1123	Pollen	Improvement of male fertility (11)	PRE-W	Mixed with honey	Oral	17	B, C
			Pith parenchyma	Memory improvement (5)	NER-N	Edible	Oral		
Arecaceae	*Nannorrhops ritchieana* (Griff.) Aitch.	Daz1 125	Fruit	Vegetable (8)	OTH-A	Edible	Oral	8	B, C
Asparagaceae	*Leopoldia comosa* (L.) Parl.	Sirmook 1235	Bulb	Anti-diarrhea (2), Bronchitis (3), Cough (1)	GAS-D, RES-R, RES-R	Mixed with food	Oral	6	B, C
Asteraceae	*Achillea eriophora* DC.	Gole bengerask 921	Flowering branches	Relaxing (4), Gastric discomfort (7), Parasite repellent (3), Anti-diarrhea (10), Menstrual disorders (2), Cramps, Febrifuge (8), Stomachache (12)	NER-N, GAS-D, GAS-D, GAS-D, GYN-X, SKE-L, OTH-A, GAS-D	Infusion, Powder	Oral	46	A
Asteraceae	*Achillea santolinoides* Lag.	Gole bengerask 918	Flowering branches	Relaxing (4), gastric discomfort (7), Parasite repellent (3), Anti-diarrhea (10), Menstrual disorders (2), Cramps, Febrifuge (8), Stomachache (12)	NER-N, GAS-D, GAS-D, GAS-D, GYN-X, SKE-L, OTH-A, GAS-D	Infusion, Powder	Oral	46	A, C
Asteraceae	*Achillea wilhelmsii* C. Koch	Gole bengerask 922	Flowering branches	Stomach ache (12), Disinfectant (1), Blood purifier (2), Carminative (4), Diuretic (1), Antispasmodic (6)	GAS-D, OTH-A, Blood-B, GAS-D, URO-U,NER-N	Decoction	Oral	26	A
Asteraceae	*Arctium lappa* L*.*	Babaadam 950	Leaf	Vertigo (3), Blood purifier (2), Antispasmodic (4), Detoxification (1), Food digestion (7), Parasite repellent (1), Kidney diseases (2)	NER-N, OTH-A, NER-N, OTH-A, GAS-D, GAS-D, URO-U	Poultice, Decoction	Oral	20	B, C
Asteraceae	*Artemisia absinthium* L.	Afsantin 930	Leaf, Flower	Intestinal parasites (5)	GAS-D	Decoction	Oral	5	A
Asteraceae	*Artemisia aucheri* Boiss.	Jaz 934	Flowering branches	Relaxing (6), Abdominal pains (27), Respiratory diseases (3), Body tonic (6), Febrifuge (25)	NER-N, GAS-D, RES-R, OTH-A, OTH-A	Decoction	Oral	68	A, C
			Flowering branches	Beauty of skin and hair (1)	DER-S	Essential oil, Aromatic water	Topical		
Asteraceae	*Artemisia persica* Boiss.	Dermene torki 931	Flowering branches	Febrifuge (44), Gastric infection (62), Stomachache (73)	OTH-A, GAS-D	Decoction	Oral	179	A, C
Asteraceae	*Artemisia scoparia* Waldst. & Kitam.	Dermeneh 932	Aerial parts	Stomachache (13)	GAS-D	Decoction	Oral	13	B, C
Asteraceae	*Artemisia sieberi* Besser	Sorkhdermon 953	Flowering branches	Anti-nausea (20), Antispasmodic, (8) Parasite repellent (18)	GAS-D, NER-N, GAS-D	Decoction	Oral	61	A
			Flowering branches	Bruise (15)	SKE-L	Decoction	Topical		
Asteraceae	*Atractylis cancellata* L*.*	Kharcharkha 940	Gum, Leaf	Vegetable (3)	OTH-A	Row, Powder	Oral	3	B, C
Asteraceae	*Calendula officinalis* L.	Gole bahari 942	Aerial parts	Carminative (2), Pancreatic cancer (1)	GAS- D, CAN-C	Infusion	Oral	8	B, C
			Aerial parts	Acne (3), Pterygium (2)	DER-S, EYE-F	Oil*,* Aromatic water	Topical, Eye drop		
Asteraceae	*Carthamus lanatus* L.	Kharzard 971	Flower	Bruise (2)	SKE-L	Poultice	Topical	2	B, C
Asteraceae	*Carthamus oxyacantha* M.Bieb.	Golrangzard 972	Leaf and flower	Purgative (1), Menstrual disorders (1), Blood purifier (1)	GAS-D, GYN-X, OTH-A	Decoction	Oral	3	B, C
Asteraceae	*Centaurea benedicta* (L.) L.	Khar moghadas 980	Flowering branches	Memory tonic (2)	NER-N	Decoction	Oral	2	B, C
Asteraceae	*Centaurea bruguierana* (DC.) Hand.-Mazz.	Gole gandom 981	Aerial parts	Anti-inflammatory (2)	SKE-L	Decoction	Oral	2	A
Asteraceae	*Cichorium intybus* L.	Kasni 925	Leaf and Flower	Jaundice (15), Liver diseases (2), Diuretic (2), Febrifuge (13), Antihypertensive (2), Laxative (55)	GAS-D, GAS-D, URO-U, OTH-A,CAR-K, GAS-D	Aromatic water, Maceration	Oral	89	A, C
Asteraceae	*Cichorium pumilum* Jacg*.*	Kasni 913	Leaf and Flower	Jaundice (14), Liver diseases (2), Febrifuge (14), Blood purifier, Antihypertensive (2), Laxative (50)	GAS-D, GAS-D, OTH-A, OTH-A, GAS-D	Aromatic water, Maceration	Oral	96	A
			Root	Appetizing (5)	OTH-A	Decoction	Oral		
Asteraceae	*Cirsium arvense* (L.) Scop.	Kangar 925	Root, Pith parenchyma	Gastric discomfort (8), Appetizing (2), Disinfectant (4), Febrifuge (7)	GAS-D, OTH-A, OTH-A, OTH-A	Decoction	Oral	21	A
Asteraceae	*Cota tinctoria* (L.) J.Gay	Babouneh 939	Flowering branches	Throat pains (4), Nervous problems (13), Common cold (14), Anti-diarrhea (20), Prostate (5)	GAS-D, NER-N, RES-R, GAS-D	Decoction	Oral	56	A, C
Asteraceae	*Cousinia congesta* Bunge.	Poloosh 945	Gum	Asthma (2)	RES-R	Decoction	Oral	2	B, C
Asteraceae	*Cyanus depressus* (M.Bieb.) Soják	Gole gandom 977	Flower	Digestive (11), Cough (1), Laxative (6)	GAS-D, RES-R, GAS-D	Decoction	Oral	18	B, C
Asteraceae	*Echinops ritrodes* Bunge	Kaloor 983	Fruit	Gastric discomfort (7)	GAS-D	Decoction	Oral	7	B, C
Asteraceae	*Glebionis coronaria* (L.) Cass. ex Spach.	Davoodi 986	Aerial parts	Blood purifier (2), Eyesight enhancement (2)	OTH-A, EYE-F	Infusion	Oral	4	B, C
Asteraceae	*Gundelia tournefortii* L.	Kangar 974	Pith parenchyma	Gastric discomfort (8), Constipation (12), Reduce Blood fat (1), Blood purifier (15)	GAS-D, GAS-D, OTH-A, OTH-A	Edible, Salad	Oral	36	B, C
Asteraceae	*Hertia angustifolia* (DC.) Kuntze	Karkich biabani 975	Leaf, Flower	Pain relief (4)	NER-N	Decoction	Oral	4	B, C
Asteraceae	*Hertia intermedia* (Boiss.) Kuntze	Karkich 973	Flowering branches	Insect bite (5), Purgative (2), Parasite repellent (1)	DER-S, GAS-D, GAS-D	Poultice, Decoction	Topical, Oral	8	A
Asteraceae	*Inula britannica* L*.*	Mosafa 979	Aerial parts	Reducing thirst (3)	OTH-A	Syrup	Oral	3	B, C
Asteraceae	*Lactuca orientalis* (Boiss.) Boiss.	Jaroo 958	Latex, Flower	Insomnia (3)	OTH-A	Decoction	Oral	3	B, C
Asteraceae	*Lactuca serriola* L.	Kahokhardar 916	Aerial parts, Latex	Bone and joint pains (1), Purgative (1)	SKE-L, GAS-D	Poultice, Decoction	Topical, Oral	2	A
Asteraceae	*Launaea acanthodes* (Boiss.) Kuntze	Goojar 918	Aerial parts	Animal parasite repellent (122), Pain relief (4)	GAS-D, NER-N	Maceration	Oral	126	B, C
Asteraceae	*Matricaria chamomilla* L.	Babounak 946	Flowering branches	Anti-inflammation (2), Anti-nausea (3)	SKE-L, GAS-D	Decoction	Oral	5	B, C
Asteraceae	*Onopordum carmanicum* (Bornm.) Bornm.	Kangar 919	Young branches	Gastric discomfort (4)	GAS-D	Decoction	Oral	4	B, C
Asteraceae	*Onopordum leptolepis* DC.	Kangar 920	Aerial parts	Urinary stone (6), Abdominal pains (9), Anti-diarrhea (8)	URO-U, GAS-D, GAS-D	Decoction	Oral	23	B, C
Asteraceae	*Rhaponticum repens* (L.) Hidalgo	Talkhe sadi 927	Aerial parts	Baby fever (2), Cancer (2)	OTH-A, CAN-C	Poultice, Decoction	Topical	4	B, C
Asteraceae	*Scorzonera mucida* "Rech.f., Aellen & Esfand.	Kalaghoo 912	Fresh leaf	Infectious wound (3)	DER-S	Poultice	Topical	3	B, C
Asteraceae	*Senecio glaucus* L.	Bangdaneh 990	Aerial parts	Chronic wound (6)	DER-S	Poultice	Topical	6	B, C
Asteraceae	*Silybum marianum* (L.) Gaertn.	Kharmaryam 995	Leaf	Fatty liver (35), Reduce blood sugar (3)	GAS-D, MET-T	Decoction	Oral	38	A, C
Asteraceae	*Sonchus asper* (L.) Hill	Shirtighak 996	Leaf	Skin rash (3)	DER-S	Poultice	Topical	3	A
Asteraceae	*Sonchus oleraceus* (L.) L.	Shirtighak 997	Leaf	Skin rash (3)	DER-S	Poultice	Topical	4	A
Asteraceae	*Tanacetum parthenium* (L.) Sch.Bip.	Babouneh 960	Aerial parts	Parasite repellent (4), Migraine (2), Anti-inflammation (10), Peptic ulcer (3), Gastritis (6)	OTH-A, NER-N, SKE-L, GAS-D, GAS-D	Decoction	Oral	25	A
Asteraceae	*Taraxacum assemanii* Boiss.	Shirdandan 998	Leaf, Flower	Liver tonic (1), Diuretic (1)	GAS-D, URO-U	Decoction	Oral	2	B, C
Asteraceae	*Taraxacum pseudocalocephalum* Soest.	Gasedak 906	Seed	Blood fat (1)	Blood-B	Decoction	Oral	1	B, C
Asteraceae	*Tragopogon graminifolius* DC.	Sheng 999	Leaf, Root	Diuretic (3), Gastrointestinal disorders (2)	URO-U, GAS-D	Decoction	Oral	5	B, C
Berberidaceae	*Berberis integerrima* Bunge	Zarch 681	Root	Reduce blood sugar (61), Animal parasite repellent (2), Hepatitis (2), Joint pains (22), Breaking bone healing (15), Leaving addiction (27)	MET-T, GAS-D, CAR-K, GAS-D, SKE-L, SKE-L	Decoction	Oral	182	A
			Leaf	Textile fiber color (9), Blood pressure (28)	OTH-A, CAR-K	Decoction	-		
			Fruit	Blood purifier (30), Heat regulation (1), Hives (3), Laxative (4)	OTH-A, OTH-A, DER-S, GAS-D	Decoction	Oral		
Biebersteiniaceae	*Biebersteinia multifida* DC.	Piche bahman 683	Root	Reinforcing sexual desire (9), Pain relief (5), Colic (3)	OTH-A, NER-N, GAS-D	Decoction	Oral	17	B, C
Bignoniaceae	*Tecomella undulata* (Sm.) Seem.	Golparak 701	Stem bark, Leaf	Skin ailments (79), Eczema (64), Reduce blood sugar (41), Urinary problems (10)	DER-S, DER-S, MET-T	Poultice, Decoction	Topical	194	A
Boraginaceae	*Anchusa azurea* Mill.	Gavzaban 710	Flower	Relaxing (4)	NER-N	Decoction, Infusion	Oral	4	A, C
Boraginaceae	*Buglossoides arvensis* (L.) I.M.Johnst.	Sangdaneh 711	Leaf, Root	Pain relief (6)	NER-N	Decoction	Oral	6	B, C
Boraginaceae	*Caccinia macranthera* (Banks & Sol.) Brand	Gavzaban 720	Flower	Relaxing (3)	NER-N	Decoction	Oral	3	A, C
Boraginaceae	*Cordia myxa* L.	Pohil 724	Fruit	Common cold (5), Appetizing (4),Throat pain (10), Eczema (2), Kidney stone (5)	RES-R, -, GAS-D, DER-S, URO-U	Poultice, Infusion	Oral, Topical	26	B, C
Boraginaceae	*Echium amoenum* Fisch. & C.A.Mey.	Golgavzaban 734	Flower	Sleeplessness (27), Relaxing (76), Anorexia (1)	NER-N, NER-N, Psy-P	Decoction	Oral	104	A, C
Boraginaceae	*Nonea caspica* (Willd.) G.Don	Gavzabanak 715	Leaf, Flower	Relaxing (2), Anorexia (1)	RES-R, NER-N, Psy-P	Decoction	Oral	5	A
Boraginaceae	*Nonea persica* Boiss*.*	Serkoee Cheskoee 712	Flower and Leaf	Relaxing (3), Heart tonic (5), Expectorant (2), Disinfectant (4),	NER-N, CAR-K, RES-R, OTH-A	Decoction	Oral	14	B, C
Boraginaceae	*Onosma stenosiphon* Boiss.	Hoochoo 716	Root	Women infection (7), Pain relief (6), Bruise (8), Wound sucker (13), Burn healing (9)	GYN-X, NER-N, DER-S, DER-S, DER-S	Poultice, Decoction	Oral, Topical	43	B, C
Boraginaceae	*Solenanthus circinatus* Ledeb.	Choobe Azar 725	Stem bark	Bruise (95)	DER-S	Poultice	Topical	95	B, C
Boraginaceae	*Trichodesma stocksii* Boiss.	Gavzaban 727	Flower	Nerve tonic (6), Respiratory ailments (1), Sore throat (1), Relaxing (1)	NER-N, RES-R, GAS-D	Decoction	Oral	9	A
Brassicaceae	*Alyssum linifolium* Stephan ex Willd.	Ghodoomeh 756	Seed	Laxative (3), Cough (2)	GAS-D, RES-R	Decoction	Oral	5	A, C
Brassicaceae	*Alyssum szovitsianum* Fisch. & C.A.Mey.	Toodari karopoo 740	Seed	Laxative (14)	GAS-D	Decoction	Oral	14	B, C
Brassicaceae	*Brassica nigra* (L.) K.Koch	Khardal 750	Root, Leaf, Seed	Memory improvement (1), Skin clarification (2)	NER-N, DER-S	Poultice, Decoction	Oral, Topical	3	B, C
Brassicaceae	*Brassica rapa* L*.*	Shalgham 749	Root	Respiratory ailments (5) ,Common cold (69)	RES-R, RES-R	Edible	Oral	74	A
Brassicaceae	*Capsella bursa-pastoris* (L.) Medik.	Kisekeshish 745	Aerial parts	Blood purifier (7)	Blood-B	Decoction	Oral	7	B, C
Brassicaceae	*Descurainia sophia* (L.) Webb. ex Prantl	Khakshir 762	Seed	Laxative (42), Disinfectant (5), Reducing thirst (7), Constipation (46), Throat infection (10), Intestinal pain (7), Blood purifier (4), Heatstroke (7), Anti-diarrhea (5)	GAS-D, GAS-D, OTH-A, -, GAS-D , GAS-D, GAS-D, OTH-A, GAS-D	Decoction, Syrup, Maceration	Oral	96	A
Brassicaceae	*Eruca vesicaria* (L.) Cav.	Mandow 755	Young stem and leaf	Body tonic (2)	OTH-A	Salad	Oral	2	B, C
Brassicaceae	*Erysimum crassicaule* (Boiss.) Boiss.	Khakshire talkh 760	Seed	Respiratory ailments (4)	RES-R	Maceration	Oral	4	A, C
Brassicaceae	*Fortuynia garcinii* (Burm.f.) Shuttlew.	Shabboo 765	Aerial parts, Laef and flower	Migraine (5), Relaxing (6), Menstrual disorders (2), Flavoring of food (4), Antispasmodic (3), Stomach tonic (6)	NER-N, NER-N, GYN-X, NER-N, GAS-D	Decoction, Infusion, Mixed with food	Oral	26	A, C
Brassicaceae	*Goldbachia laevigata* (M.Bieb.) DC.	Nakhonak 780	Seed	Antimicrobial (1)	OTH-A	Decoction	Oral	1	B, C
Brassicaceae	*Lepidium draba* L.	Mookoo 759	Leaf	Eczema (4), Reduce rheumatic pain (7), Diuretic (8), Gastritis (4), Stomach acidification (4), Cough (8), Flavoring of food (16), Anemia (3)	DER-S, SKE-L, URO-U, GAS-D, GAS-D, RES-R, OTH-A, Blood-B	Poultice, Decoction	Topical, Oral	54	B, C
Brassicaceae	*Lepidium latifolium* L*.*	Tarantizak 770	Aerial parts	Pickle (9), Body tonic (3)	OTH-A , OTH-A	Edible	Oral	12	B, C
Brassicaceae	*Lepidium sativum* L*.*	Shahi 776	Leaf	Muscle cramps (2), Reduce rheumatic pain (3)	SKE-L, SKE-L, SKE-L, SKE-L	Decoction	Oral	5	B, C
Brassicaceae	*Raphanus caudatus* L*.*	Torobcheh 784	Root	Digestive (6), Urinary problems (2)	GAS-D, URO-U	Vegetable, Decoction	Oral	8	B, C
Brassicaceae	*Sisymbrium irio* L.	Khakshir 763	Seed	Laxative, Constipation	GAS-D, GAS-D	Infusion	Oral		A
Brassicaceae	*Isatis tinctoria* L.	Vasmeh 783	Leaf	Hair tonic and hair color (24)	DER-S	Powder mixed with water	Topical	24	A, C
Brassicaceae	*Thlaspi perfoliatum* L*.*	Kisehchoochan 785	Seed	Diuretic (5)	URO-U	Decoction	Oral	5	B, C
Cannabaceae	*Cannabis sativa* L*.*	Kanaf 1240	Seed, Leaf, flowering branches	Urinary problems (3), Sleeplessness (2), Nervous system tonic (7), Relaxing (2)	URO-U, NER-N, NER-N, NER-N	Decoction	Oral	14	B, C
Campanulaceae	*Campanula kermanica* (Rech.f., Aellen & Esfand.) Rech.f.	Gole ghifoo 1325	Flower and leaf	Cough (4)	RES-R	Decoction	Oral	4	B, C
Capparidaceae	*Capparis spinosa* L*.*	Dak 634	Fruit, Leaf	Liver diseases (1), Anemia (1), Joint pains (3), Antimicrobial (12), Pickle (7), Eczema (16)	RES-R, OTH-A, SKE-L, OTH-A, OTH-A, DER-S	Decoction, Poultice	Oral , Topical	40	A
Caprifoliaceae	*Lomelosia olivieri* (Coult.) Greuter & Burdet	Sarbanafsheh talkh 1331	Flower	Diarrhea (5), Joint pans (4)	GAS-D, SKE-L	Decoction, Poultice	Topical, Oral	9	B, C
Caprifoliaceae	*Scabiosa candollei* DC.	Talhkou 1332	Flower	Anti-diarrhea (8), Abdominal pains (5), Bone and joint pains (2)	GAS-D, GAS-D, SKE-L	Decoction, Poultice	Topical	16	A
Caprifoliaceae	*Scabiosa flavida* Boiss. & Hausskn.	Sarbanafsheh talkh 1334	Flower	Anti-diarrhea (4), Joint pans (3)	GAS-D, SKE-L	Decoction, Poultice	Topical, Oral	7	B, C
Caprifoliaceae	*Valeriana ficariifolia* Boiss.	Alafe gorbe 1337	Root, Rhizome	Relaxing (5)	NER-N	Decoction	Oral	5	B, C
Caryophyllaceae	*Dianthus crinitus* Sm.	Ghernefel 612	Seed	Toothache (17), Breath freshener (2)	OTH-A, RES-R	Decoction, Poultice	Oral, Topical	19	B, C
Caryophyllaceae	*Dianthus orientalis* Adams	Mikhak 611	Leaf, Flower	Toothache (24), Breath freshener (2), Headache (18), Nerve pain (27)	OTH-A, RES-R, NER-N, NER-N	Poultice, Decoction	Oral, Topical	71	A
Caryophyllaceae	*Herniaria hirsuta* L.	Fetgh 613	Aerial parts	Burn wound healing (5)	DER-S	Poultice	Topical	5	B, C
Cleomaceae	*Cleome coluteoides* Boiss.	Alafe maar 1350	Leaf, Flower and Fruit	Diuretic (5), Laxative (2), Anti-nausea (2), psoriasis (2)	URO-U, GAS-D, GAS-D, DER-S	Decoction, Poultice	Oral	11	B, C
Colchicaceae	*Colchicum schimperi* Janka ex Stef.	Hasratoo 452	Root	Wart treatment (5), Joint pains (6), Reduce the pain of gout disease (4)	DER-S, SKE-L, NER-N	Poultice	Topical	15	B, C
Convolvulaceae	*Convolvulus arvensis* L.	Pichak 791	Leaf, Flower, Seed	Gastric discomfort (12), Wound healing (3), Asthma (2)	GAS-D, DER-S	Decoction, Poultice	Oral, Topical	17	A
Convolvulaceae	*Cressa cretica* L.	Alaf mourcheh 790	Aerial parts	Antifungal (4), Antibacterial (14)	OTH-A, OTH-A	Poultice	Topical	18	A
Convolvulaceae	*Cuscuta epithymum* (L.) L.	Ses 792	Aerial parts	Diuretic (2), Jaundice (2)	URO-U, GAS-D	Decoction	Oral	4	A, C
Cucurbitaceae	*Citrullus colocynthis* (L) Schrad.	Gelgenjak 890	Fruit, Seed, Root	Reduce blood sugar (91), Reduce rheumatic pain (3), Scorpion bite (6), Chronic ulcers (5), Antihypertensive (4), Febrifuge (2), Bone and joint pains (8)	MET-T, DER-S, DER-S, OTH-A, CAR-K, DER-S, SKE-L	Poultice, powder	Topical, Oral	119	A
Cucurbitaceae	*Cucumis sativus* L.	Kheyar 889	Aerial parts	Laxative (13)	GAS-D	Decoction	Oral	13	A, C
Cucurbitaceae	*Cucurbita moschata* Duchesne	Kadoohalvaee 891	Seed	Prostate (6)	URO-U	Decoction	Oral	6	A, C
Cucurbitaceae	*Cucurbita pepo* L.	Kadoo 885	Fruit	Blood fat (8), Constipation (4)	Blood-B, GAS-D	Edible	Oral	12	A, C
Cupressaceae	*Juniperus excelsa* M.Bieb*.*	Avors 203	Fruit	Common cold (14), Hair tonic and hair color (3), Freshener body (6), Skin rash (31), Wound healing (12)	RES-R, DER-S, OTH-A, DER-S, DER-S	Powder, Bath, Decoction, Poultice	Topical, Oral	143	B
			Leaf	Pest Control (15), Algae Pool Control (3), Wood corrosion (10), Pain relief (4)	OTH-A, OTH-A, OTH-A, NER-N	Dressing, Powder, Decoction	Topical, Oral		
			Leaf and fruit	Stomach tonic (7), Appetizing (5), Reduce rheumatic pain (11)	GAS-D, OTH-A, SKE-L	Decoction, Poultice	Oral, Topical		
Elaeagnaceae	*Elaeagnus angustifolia* L.	Senjed 544	Seed	Joint pains (26), Anti-diarrhea (48), Peptic ulcer (10)	SKE-L, GAS-D, GAS-D	Poultice, Powder	Topical, Oral	90	A
			seed	Health tonic (6)	OTH-A	Oil	Oral		
Ephedraceae	*Ephedra distachya* L.	Khimouk 182	Young branches	Stomachache (12), Relaxing (8), Peptic ulcer (62), Stomach burning, (22) Traditional tannery (10), Relaxing (1)	GAS-D, NER-N, GAS-D, GAS-D, GAS-D, NER-N	Decoction, Infusion	Oral	115	B, C
Ephedraceae	*Ephedra foliata* Boiss. ex C.A.Mey.	Khimouk 180	Young branches	Stomachache (2), Relaxing (3), Peptic ulcer (3), Relaxing (1)	GAS-D, NER-N, GAS-D, NER-N	Decoction, Infusion	Oral	9	B, C
Ephedraceae	*Ephedra major* Host	Alijoon 185	Young branches, Fruit	Respiratory ailments (4), Cough (4), Common cold (5), Pain relief (24), Relaxing (1)	RES-R, RES-R, RES-R, NER-N, NER-N	Decoction	Oral	53	A
			Young branches, Fruit	Traditional tannery (15)	OTH-A	Decoction	-		
Ephedraceae	*Ephedra intermedia* Schrenk & C.A.Mey.	Khimouk 179	Young branches	Common cold (5), Stomachache (7), Weight loss (36), Peptic ulcer (50), Traditional tannery (15), Relaxing (1)	RES-R, GAS-D, OTH-A, GAS-D, OTH-A, NER-N	Decoction, Infusion	Oral	114	B, C
Ephedraceae	*Ephedra pachyclada* Boiss*.*	Hoome nar 186	Young branches, Fruit	Cramp (9), Food coloring (10)	SKE-L, OTH-A	Decoction, Infusion	Oral	19	A, C
Ephedraceae	*Ephedra strobilacea* Bunge	Khimook 189	Young branches, Fruit	Pain relief (37), Gastric discomfort (53), Relaxing (1)	NER-N, GAS-D, NER-N	Decoction	Oral	91	A
Euphorbiaceae	*Euphorbia bussei* Pax	Shirsag 470	Young leaf	Treatment of Blister (5), Skin stimulant (3), Reduced vision (3), Anemia (2)	DER-S, DER-S, EYE-F, Blood-B	Decoction, Poultice	Oral, Topical	13	A, C
Euphorbiaceae	*Euphorbia granulata* Forssk*.*	Shirbeng 472	Latex	Eczema (2)	DER-S	Latex	Topical	2	B, C
Euphorbiaceae	*Euphorbia helioscopia* L*.*	Shirbeng 484	Leaf, Seed	Joint pains (3), Skin rash (3), Reduce rheumatic pain (2)	SKE-L, DER-S, SKE-L	Decoction, Poultice	Topical, Oral	8	A
Euphorbiaceae	*Euphorbia peplus* L*.*	Alafe zegi l475	Latex	Eczema (2)	DER-S	Latex	Topical	2	B, C
Euphorbiaceae	*Euphorbia serpens* Kunth	Gazeroo 477	Latex	Eczema (10)	DER-S	Latex	Topical	10	B, C
Euphorbiaceae	*Euphorbia turcomanica* Boiss*.*	Farfeyeon 478	Latex	Eczema (3)	DER-S	Latex	Topical	3	B, C
Euphorbiaceae	*Ricinus communis* L*.*	Kenton 499	Seed	Laxative (3), Skin patches (2), Hair tonic (5), Disinfectant (4)	GAS-D, DER-S, DER-S, OTH-A	Poultice, Oil	Topical	13	A
Fabaceae	*Alhagi maurorum* Medik.	Adoor 675	Aerial parts	Urinary stone (13), Hemorrhoid (2), Reduce rheumatic pain (1)	URO-U, CAR-K, SKE-L	Decoction, Poultice	Topical, Oral	15	A, C
Fabaceae	*Alhagi pseudalhagi* (M. Bieb.) Desv. ex B. Keller & Shap.	Adoor 674	Aerial parts	Urinary stone (13), Child jaundice (4)	URO-U, GAS-D	Aromatic water, Decoction	Oral	17	A
Fabaceae	*Astracantha lateritia* (Boiss. & Hausskn.) Podlech	Khar 640	Stem	Hair tonic (22), Eczema (45)	DER-S, DER-S	Gel	Topical	67	B, C
Fabaceae	*Astragalus eremophilus* Boiss*.*	Gavan 643	Seed	Reinforcing sexual desire (3), Asthma (5), Preventing of the Blood coagulation (2), Expectorant (5)	OTH-A, RES-R, Blood-B , RES- R	Decoction	Oral	15	A
Fabaceae	*Astragalus fasciculifolius* subsp.*arbusculinus* (Bornm. & Gauba) Tietz	Anzaroot 645	Gum, Stem bark	Ear infection (3), Earache (2), Cough (3), Stomachache (5), Livestock parasite (6), Common cold (4), Detoxification (5), Foot-and-mouth disease of livestock (10), Wart (6), Eye ache (2)	Ear-H , Ear-H, RES-R, GAS-D, GAS-D, RES-R, OTH-A, GAS-D, DER-S, EYE-F	Poultice, Decoction	Oral, Topical	46	B, C
Fabaceae	*Astragalus gossypinus* Fisch.	Gavan 641	Gum, Stem bark	Traditional kohl (67), Hair beauty (15), Hair tonic (15)	DER-S, DER-S, DER-S	Gel, Poultice	Topical	97	B, C
Fabaceae	*Astragalus ovoideus* Širj. & Rech.f.	Margin 685	Gum	Anti-stress (4), Relaxing (4)	NER-N, NER-N	Decoction	Oral	8	B, C
Fabaceae	*Cercis siliquastrum* L*.*	Argavan 646	Leaf, Stem bark	Gastric discomfort (3), Expectorant (2)	GAS-D, RES-R	Decoction, Infusion	Oral	5	B, C
Fabaceae	*Cicer arietinum* L.	Nokhod kermani 659	Seed	Hair tonic (2), Diuretic (3)	DER-S, URO-U, CAN-C	Decoction, Poultice	Oral, Topical	5	A, C
Fabaceae	*Cicer kermanense* Bornm.	Nokhod-e kermani 660	Fruit	Hair tonic (2), Diuretic (3), Menstrual regulation (2)	DER-S, URO-U, GYN-X	Decoction, Poultice	Topical, Oral	7	A
Fabaceae	*Colutea persica* Boiss.	Feh 652	Stem bark	Wound healing (3)	DER-S	Poultice	Topical	3	B, C
Fabaceae	*Dalbergia sissoo* DC.	Jag 653	Stem bark	Abortion (2), Expectorant (7), Anti-parasitic (2), Burn healing (2), Anti-nausea (2), Reducing thirst (1)	PRE-W, RES-R, GAS-D, DER-S, GAS-D, GAS-D	Decoction, Poultice	Oral, Topical	16	A
Fabaceae	*Genista tinctoria* L*.*	Rangineh 654	Aerial parts	Anti-diarrhea (2), Gastric discomfort (4), Abdominal pains (3), Constipation (2)	GAS-D, GAS-D, GAS-D, GAS-D	Decoction	Oral	11	A
Fabaceae	*Glycyrrhiza glabra* L.	Matki 650	Root	Stomach ulcers (28), Aphthous ulcer (83), Expectorant (9), Breaking bone healing (14), Prostate (4)	GAS-D, GAS-D, RES-R, SKE-L, URO-U	Decoction, Poultice	Oral	138	A, C
Fabaceae	*Lathyrus sativus* L.	Karoo 661	Arial part	Laxative (5), Common cold (2), Fatty liver (2), Jaundice (3), Eczema (2)	GAS-D, RES-R, GAS-D, GAS-D, DER-S	Edible, Poultice	Oral, Topical	14	B, C
Fabaceae	*Lens culinaris* Medik.	Adas 663	Seed	Body tonic (10)	OTH-A	Edible	Oral	10	A, C
Fabaceae	*Medicago sativa* L.	Yonjeh 665	Young Leaf	Intestinal parasites (1), Eyesight tonic (7), Appetizing (2), Anemia (8)	GAS-D, EYE-F, OTH-A, OTH-A	Edible	Oral	28	A
			Root	Stomach tonic (3), Gastric discomfort (3), Leaving addiction (2), Reduce blood sugar (2)	GAS-D, GAS-D, NER-N, MET-T	Decoction	Oral		
Fabaceae	*Melilotus officinalis* (L.) Pall.	Kalilalmolk 697	Leaf and young stem	Common cold (4), Diuretic (2), Relaxing (9), Antispasmodic (3)	RES-R, URO-U, NER-N, NER-N	Edible	Oral	18	B, C
Fabaceae	*Onobrychis altissima* Grossh.	Esperes 670	Leaf, Flower	Jaundice (7)**,** Appetizing (2)	GAS-D, OTH-A	Decoction	Oral	9	A
Fabaceae	*Ononis spinosa* L.	Kharkhar 668	Root	Inflammation of the urinary tract (6), Diuretic (6)	URO-U, URO-U	Decoction	Oral	12	B, C
Fabaceae	*Phaseolus vulgaris* L*.*	Loobia sabz 669	Fruit	Cardiovascular diseases (5), Diuretic (2), Cancer (1)	CAR-K, URO-U, CAN-C	Mixed with food	Oral	8	B, C
Fabaceae	*Prosopis cineraria* (L.) Druce	Kahoor 655	Latex	Eczema (10)	DER-S	Latex of burning stem	Topical	26	B, C
			Stem bark	Traditional tannery (16)	OTH-A	Decoction	-		
Fabaceae	*Prosopis farcta* (Banks & Sol.) J.F.Macbr.	Kahoorak 684	Dried Fruit	Antihistamine (2), Preventing of nose bleeding (2)	RES-R, RES-R	Decoction	Oral	4	B, C
Fabaceae	*Sophora alopecuroides* L*.*	Talkheh 687	Whole plant	Antihypertensive (1), Antibacterial (2), Constipation (2), Pain relief (1)	CAR-K, OTH-A, GAS-D, NER-N	Decoction	Oral	6	B, C
Fabaceae	*Sophora mollis* (Royle) Baker	Talkheh 688	Seed	Antihypertensive (1), Antibacterial (2), Constipation (2), Pain relief (1)	CAR-K, OTH-A, GAS-D, NER-N	Mixed with food	Oral	6	B, C
Fabaceae	*Sophora pachycarpa* C.A.Mey.	Talkheh 690	Seed	Antihypertensive (1), Antibacterial (2), Constipation (2), Pain relief (1)	CAR-K, OTH-A, GAS-D, NER-N	Mixed with food	Oral	6	B, C
Fabaceae	*Taverniera cuneifolia* (Roth) Ali	Lati 614	Leaf	Wound healing (9)	DER-S	Poultice	Topical	9	B, C
Fabaceae	*Taverniera nummularia* DC.	Daf 615	Leaf	Wound healing (9)	DER-S	Poultice	Topical	9	B, C
Fabaceae	*Tragacantha fasciculifolia* (boiss.) Kuntze	Khaar 680	Stem and leaf	Hair tonic (3), Gingival inflammation (5)	DER-S, GAS-D	Gum, Powder	Topical, Oral	8	B, C
Fabaceae	*Trifolium repens* L.	Shabdare sefid 693	Aerial parts	Blood purifier (3), Cough (2), Cardiovascular disorders (1), Anti-diarrhea (3), Digestive (4), Burn healing (6)	OTH-A, RES-R, CAR-K, GAS-D, GAS-D, DER-S	Decoction, vegetable	Oral	9	A, C
Fabaceae	*Trigonella foenum-graecum* L.	Shanbalileh 694	Leaf and young stem	Antihypertensive (6), Reduce blood sugar (5)	CAR-K, MET-T	Decoction	Oral	21	A
Fabaceae	*Vicia faba* L*.*	Bagla 689	Seed	Reduce the blood fat (3), Constipation (4)	Blood-B, GAS-D	Food	Oral	7	A
Fabaceae	*Vicia sativa* L*.*	Mash 617	Seed	Anti-diarrhea (2), Diuretic (1), Gumboil (2)	GAS-D, URO-U, GAS-D	Eat as food	Oral	5	A
Fabaceae	*Lens culinaris* Medik.	Adas 630	Seed	Anti-diarrhea (2), Diuretic (1), Gumboil (2)	GAS-D, URO-U, GAS-D	Eat as food	Oral	5	A
Geraniaceae	*Erodium cicutarium* (L.) L'Hér.	Soozan Kalaghoo 1411	Aerial parts	Antibacterial (2), Wound healing (5), Anti-diarrhea (3), Intestinal infection (4)	OTH-A, DER-S, GAS-D, GAS-D	Decoction, Poultice	Oral	14	B, C
Geraniaceae	*Geranium rotundifolium* L*.*	Soozani 1412	Bulb	Intestinal infection (3), Anti-diarrhea (1)	GAS-D, GAS-D	Decoction	Oral	4	A, C
Gentianaceae	*Centaurium pulchellum* (Sw.) Druce	Ghontorionasa 1413	Aerial parts	Febrifuge (4), Body tonic (3), Carminative (3)	OTH-A, OTH-A, GAS-D	Decoction	Oral	10	B, C
Gisekiaceae	*Gisekia pharnaceoides* L.	Bargdyereii 1416	Aerial parts	Remove skin bur (5)	DER-S	Dressing	Topical	5	A
Hypericaceae	*Hypericum perforatum* L.	Raee 859	Leaf and flower	Burn wound (3)	DER-S	Poultice	Topical	3	A, C
Iridaceae	*Iris germanica* L.	Zanbagh 314	Aerial parts	Antifungal (2), Reduce rheumatic pain (2)	OTH-A, SKE-L	Decoction	Oral	4	B, C
Iridaceae	*Moraea sisyrinchium* (L.) Ker Gawl.	Zanbagh 317	Aerial parts	Cough (3), Common cold (5)	RES-R, RES-R	Decoction	Oral	8	A, C
Juglandaceae	*Juglans regia* L.	Gerdoo 1450	Leaf	Beans pest (12), Arthritis (1), treatment of Secretions of the womb (2)	OTH-A, SKE-L, GYN-X	Powder, Decoction	Topical	16	A, C
			Root bark	Tooth germ (2)	GAS-D	Tooth brush	Oral		
Lamiaceae	*Ajuga chamaecistus* Ging. ex Benth.	Sameesk 811	Leaf and flowering branched	Febrifuge (5), Antihypertensive (4), Kidney disorders (2), Antifungal (1)	OTH-A, CAR-K, URO-U, OTH-A	Vegetable	Oral	12	B, C
Lamiaceae	*Clinopodium graveolens* (M.Bieb.) Kuntze.	Melangoo 813	Fruit, Seed, Aerial parts	Detoxification (7), Peptic ulcer (6), Dry cough (8), Expectorant (8)	OTH-A, GAS-D, RES-R, RES-R	Vegetable, mixed with food, Decoction	Oral	29	B, C
Lamiaceae	*Dracocephalum polychaetum* Bornm.	Zarab 834	Leaf, flowering branches	Reduce rheumatic pain (2), Stomachache (34), Toothache (15), Headache (12), Reduce blood sugar (8), Anti-diarrhea (26), Detoxification (33), Body tonic (37), Relaxing (9), Back pain (23), Blood purifier (17)	SKE-L, GAS-D, GAS-D, NER-N, MET-T, GAS-D, OTH-A, OTH-A, NER-N, SKE-L, CAR-K	Decoction, Infusion	Oral	216	B
Lamiaceae	*Lallemantia royleana* (Benth.) Benth*.*	Malangoo 821	Fruit	Carminative (3), Cough (4), Constipation (4), Expectorant (3), Vermicide (5), Dysentery (3)	GAS-D, RES-R, GAS-D, RES-R, GAS-D, GAS-D	Decoction	Oral	22	A
Lamiaceae	*Lamium album* L*.*	Gazaneh 816	Flowering branches, Root	Anti-diarrhea (2), Wound healing (2), Reduce rheumatic pain (3)	GAS-D, DER-S, SKE-L	Decoction, Poultice	Oral, Topical	*7*	A
Lamiaceae	*Lavandula angustifolia* Mill*.*	Ostokhodoos 823	Leaf	Bone and joint pains (20), Reduce rheumatic pain (12), Relaxing (10)	SKE-L, SKE-L, NER-N	Decoction	Oral	62	A, C
Lamiaceae	*Leonurus cardiaca* L*.*	Dom shir 825	Leaf	Cardiac distress (4)	CAR-K	Decoction	Oral	4	A
Lamiaceae	*Marrubium vulgare* L.	Boogandoo 820	Leaf and flower	Bone and joint pains (4), Reduce blood sugar (7), Antibacterial (1), Reinforcing sexual desire (3), Prostate (4), Anti-diarrhea (6), Women diseases (3)	SKE-L, MET-T, OTH-A, OTH-A, URO-U, GAS-D, GYN-X	Decoction	Oral, Topical	28	B, C
Lamiaceae	*Melissa officinalis* L*.*	Badranjbooye 830	Foliage	Bone and joint pains (12), Appetizing (10), Headache (5), Dizziness (6)	SKE-L, -, NER-N, NER-N	Decoction	Oral	33	A, C
Lamiaceae	*Mentha longifolia* (L.) L.	Poodeneh 827	Leaf and Flower	Stomach ache (31), Edible (44), Common cold (6), Anti-diarrhea (30), Carminative (14), Cough (6), Gastrointestinal pains (27), Antispasmodic (3)	GAS-D, -, RES-R, GAS-D, GAS-D, GAS-D, NER-N,	Edible, Decoction	Oral	161	A
Lamiaceae	*Mentha spicata* L*.*	Pooneh sonbolehee 832	Leaf and flower	Anti-diarrhea (10), Carminative (5), Antispasmodic (6), Pain relief (7)	GAS-D, GAS-D, GAS-D, NER-N	Infusion, Mixed with food, Aromatic water	Oral	28	A
Lamiaceae	*Nepeta bornmuelleri* Hausskn. ex Bornm*.*	Poodeneh 837	Aerial parts	Relaxing (4), Anti-diarrhea (12), Carminative (2)	NER-N, GAS-D	Decoction	Oral	18	A
Lamiaceae	*Nepeta bracteata* Benth.	Zoofa 840	Flowering branches	Disinfectant (9), Common cold (15)	OTH-A, RES-R	Decoction, Powder	Oral	24	A
Lamiaceae	*Nepeta cataria* L*.*	Nana 841	Leaf	Cough (17), Febrifuge (22), Colic (18), Stomachache (42), Edible (62), Common cold (35), Anti-diarrhea (68), Carminative (19), Cough (9), Antispasmodic (7), Gastrointestinal pains (31)	RES-R, OTH-A, GAS-D, GAS-D, -, RES-R, GAS-D, GAS-D, RES-R, GAS-D, GAS-D	Infusion, Mixed with food, Aromatic water	Oral	328	A
Lamiaceae	*Nepeta daenensis* Boiss.	Pooodneh 842	Leaf and flowering branched	Antimicrobial (2), Stomachache (5), Anti-diarrhea (14)	OTH-A, GAS-D, GAS-D	Aromatic water	Oral	21	A
Lamiaceae	*Lophanthus dschuparensis* (Bornm.) Levin	Pooodneh 843	Leaf and flowering branched	Antimicrobial (2), Stomachache (5), Anti-diarrhea (14)	OTH-A, GAS-D, GAS-D	Aromatic water	Oral	21	B, C
Lamiaceae	*Nepeta glomerulosa* Boiss.	Badrange golmowroo 844	Aerial parts	Disinfectant (6)*,* Joints pain (2)	OTH-A, SKE-L	Decoction, Powder	Oral	8	A
Lamiaceae	*Nepeta ispahanica* Boiss.	Gole Zoofa 845	Leaf, Flower	Anti-inflammatory (6), Antifungal (5), Antispasmodic (3)	SKE-L, OTH-A, NER-N	Aromatic water, Decoction	Oral	14	A
Lamiaceae	*Nepeta rivularis* Bomm.	Pooodneh 846	Leaf and flowering branched	Antimicrobial (1), Stomachache (7), Anti-diarrhea (15)	OTH-A, GAS-D, GAS-D	Aromatic water	Oral	23	A
Lamiaceae	*Nepeta saccharata* Bunge.	Pooodneh 847	Leaf and flowering branched	Antimicrobial (2), Stomachache (5), Anti-diarrhea (14)	OTH-A, GAS-D, GAS-D	Aromatic water	Oral	21	A
Lamiaceae	*Nepeta supina* Steven	Makhleseh 848	Leaf and flowering branched	Anti-nausea (7)	GAS-D	Aromatic water	Oral	7	A, C
Lamiaceae	*Nepeta teucriifolia* Willd.	Poodeneh 837	Leaf and flowering branched	Antimicrobial (2), Stomachache (5), Anti-diarrhea (14), Anti-nausea (8), Gastrointestinal pains (4), Carminative (3)	OTH-A, GAS-D, GAS-D, GAS-D, GAS-D, GAS-D	Aromatic water	Oral	36	A
Lamiaceae	*Ocimum basilicum* L.	Reyhan 835	Aerial parts	Sore throat (2), Common cold (8), Flavoring of food (16), Digestive (5), Asthma (2)	OTH-A, RES-R, -, GAS-D, RES-R	Edible, decoction	Oral	33	A
Lamiaceae	*Origanum vulgare* L.	Mirzangoo 810	Aerial parts	Carminative (2), Diuretic (3), Disinfectant (3), Flavoring of food (5)	GAS-D, URO-U, OTH-A, OTH-A	Decoction, Poultice, mixed with food	Oral, Topical	13	A, C
Lamiaceae	*Rydingia persica* (Burm.f.) Scheen &V.A.Albort	Golder 822	Leaf, Flower	Reduce blood sugar (26), Liver diseases (10), Leaving addiction (28), Bone and joints pains (69), Bone tonic (68), Body tonic (52), Relaxing (1)	MET-T,GAS-D, NER-N, SKE-L, SKE-L, OTH-A, NER-N	Decoction	Oral	254	B, C
Lamiaceae	*Salvia compressa* Vent.	Morporzou 806	Aerial parts	Stomachache (47), Anti-diarrhea (25), Gastric discomfort (20), Women infection (18)	GAS-D, GAS-D, GAS-D, GYN-X	Decoction	Oral	110	A
Lamiaceae	*Salvia leriifolia* Benth.	Nowroozak 807	Foliage	Carminative (5), Stomach tonic (9)	GAS-D, GAS-D	Decoction	Oral	14	A, C
Lamiaceae	*Salvia macrosiphon* Boiss*.*	Mooreshk 812	Seed	Menstrual disorders (13), Leaving addiction (16), Anti-diarrhea (5), Antibacterial (4), Carminative (2), Reduce blood sugar (4), Wound healing (7), Blood purifier (1), Respiratory infection (2), Expectorant (3)	GYN-X, NER-N, GAS-D, OTH-A, GAS-D, MET-T, DER-S, CAR-K, RES-R, RES-R	Decoction, Poultice	Oral, Topical	56	A
Lamiaceae	*Salvia mirzayanii* Rech.f*.* & Esfand*.*	Mourporzoo 814	Leaf and young branches	Stomachache (54), Leaving addiction (11), Anti-diarrhea (46), Women infection (6)	GAS-D, NER-N, GAS-D, GYN-X	Decoction, Poultice	Oral	117	A
Lamiaceae	*Stachys lavandulifolia* Vahl	Gole moureshk 831	Leaf	Antifungal (5)	OTH-A	Decoction	Oral	5	A, C
Lamiaceae	*Stachys inflata* Benth.	Gole moorshak 805	Leaf and flower	Febrifuge (4), Body tonic (4), Anti-inflammatory (4), Respiratory ailments (2)	OTH-A, OTH-A, SKE-L, RES-R	Decoction	Oral	14	A
Lamiaceae	*Teucrium polium* L.	Kalpooreh 819	Leaf and young branches	Menstrual disorders (17), Bone pain (21), Child diarrhea (58), Stomachache (70), Febrifuge (34), Carminative (15), Reduce rheumatic pain (2), Anti-nausea (4), Anti-diarrhea (16), Reduce blood sugar (3), Gastrointestinal infection (20)	GYN-X, SKE-L, GAS-D, GAS-D, OTH-A, GAS-D, SKE-L, GAS-D, GAS-D, GAS-D, GAS-D	Decoction	Oral	260	A
Lamiaceae	*Teucrium scordium* L*.*	Maryamgoli 815	Flowering branches	Stomachache (17)	GAS-D	Decoction	Oral	17	A,
Lamiaceae	*Thymus fedtschenkoi* Ronneger.	Avishan 828	Leaf and flower	Cough (102), Expectorant (31), Common cold (55), Antibacterial (20), Sore throat (32)	RES-R, RES-R, RES-R, RES-R, OTH-A	Decoction	Oral	240	A
Lamiaceae	*Zataria multiflora* Boiss.	Avishane-shirazi 807	Leaf and young stem	Cough (104), Expectorant (36), Common cold (64), Sore throat (43), Antibacterial (32)	RES-R, RES-R, RES-R, RES-R, OTH-A	Decoction	Oral	281	A
Lamiaceae	*Ziziphora clinopodioides* Lam*.*	Aghalaleh 804	Leaf and flower	Flavoring of food (30), Common cold (20), Nerve tonic (13), Relaxing (15)	OTH-A, RES-R, NER-N, NER-N	Edible, Decoction	Oral	83	A
Lamiaceae	*Ziziphora tenuior* L*.*	Kakooti 803	Aerial parts	Gastrointestinal pains (18), Body tonic (9), Stomach tonic (5), Flavoring of food (30)	GAS-D, OTH-A, GAS-D	Decoction	Oral	62	A
Liliaceae	*Tulipa biflora* Pall.	Laleh 1010	Bulb	Cough (6)	RES-R	Powder	Oral	6	A
Linaceae	*Linum album* Ky. ex Boiss*.*	Gole-sefidoo 604	Seed	Prostate (3), Weight loss (2), Anorexia (4)	URO-U, OTH-A, Psy-P	Infusion	Oral	14	B, C
Linaceae	*Linum usitatissimum* L.	Ketan 606	Seed	Prostate (3), Weight loss (2), Anorexia (4)	URO-U, OTH-A, Psy-P	Infusion	Oral	9	A, C
Lythraceae	*Lawsonia inermis* L.	Hana 1020	Leaf	Jaundice (63), Fingernail and hair tonic (57), Eczema (18), Burn scar (21)	GAS-D, DER-S, DER-S, DER-S	Bath, Decoction, Poultice	Oral, Topical	164	A
			Root	Diuretic (3), Bronchitis (2)	DER-S, RES-R	Decoction	Oral		
Lythraceae	*Punica granatum* L.	Anar 1021	Peel skin	Stomach ulcers (35), Wound healing (32), Sore throat (10)	GAS-D, DER-S, OTH-A	Poultice, Decoction, Powder	Oral, Topical	77	A
Malvaceae	*Althaea aucheri* Boiss.	Khatmi 584	Leaf	Laxative (30), Cough (3), Antihypertensive (7), Jaundice (15), Skin discomfort (8)	GAS-D, RES-R, CAR-K, GAS-D, DER-S	Maceration	Oral	63	A
Malvaceae	*Grewia tenax* (Forssk.) Fiori*.*	Pootoorak 570	Stem	Cough (2)	RES-R	Decoction	Oral	2	B, C
Malvaceae	*Hibiscus sabdariffa* L*.*	Chay torsh 571	Leaf	Antihypertensive (10)	CAR-K	Decoction	Oral	10	A, C
Malvaceae	*Malva microcarpa* Pers.	*Khatmi* 574	Seed, Leaf	Pain relief (13), Anti-inflammatory (10), Disinfectant (3), Jaundice (19), Infectious wound (6)	NER-N, SKE-L, OTH-A, GAS-D, DER-S	Decoction, Maceration, Poultice	Oral, Topical	51	A
Malvaceae	*Malva neglecta* Wallr.	Khatmi sefid 575	Seed, Leaf	Diuretic (5), Anti-inflammatory (3)	URO-U, SKE-L	Maceration	Oral	8	A, C
Malvaceae	*Malva sylvestris* L.	Khatmi 577	Seed, Leaf	Heatstroke (4), Febrifuge (4), Mouth and throat protuberance (5), Cough (6),	OTH-A, OTH-A, GAS-D, RES-R,	Maceration	Oral	19	A
Menispermaceae	*Cocculus pendulus* (J.R.Forst. & G.Forst.) Diels	Pichakoo 1030	Leaf	Febrifuge (9)	OTH-A	Decoction	Oral	9	B, C
Moraceae	*Ficus carica* L.	Anjir 1040	Fruit, Leaf	Constipation (5), Memory improvement (3), Common cold (5), Sore throat (6)	GAS-D, NER-N, RES-R, OTH-A	Infusion	Oral	19	B, C
Moraceae	*Ficus johannis* Boiss*.*	Anjir-e dalmi 1042	Leaf	Stomach tonic (15)	GAS-D	Decoction	Oral	15	B, C
Moraceae	*Morus alba* L.	Toot-e sefid 1043	Fruit	Reduce blood sugar (2), Relaxing (2), Diuretic (1)	MET-T, NER-N, URO-U	Edible	Oral	11	A
			Leaf	Febrifuge (3)	OTH-A	Edible	Oral		
			Stem bark	Constipation (3)	GAS-D	Edible	Oral		
Moraceae	*Morus nigra* L.	Shahtoot 1044	Fruit	Common cold (8), Sore throat (7), Urinary infection (2)	RES-R, OTH-A, URO-U	Syrup	Oral	17	A
Myrtaceae	*Eucalyptus camaldulensis* Dehnh.	Kalitus 1700	Leaf	Common cold (28), Respiratory ailments (22), Disinfectant (3)	RES-R, RES-R, OTH-A	Decoction, Poultice	Oral, Topical	54	A
Myrtaceae	*Myrtus communis* L.	Moordaneh 1050	Leaf, Seed	Common cold (14), Relaxing (3), Removing the armpit odor (5)	RES-R, NER-N, OTH-A	Decoction	Oral, Topical	22	A
Myrtaceae	*Syzygium cumini* (L.) Skeels	Jam1051	Ash of Leaf and seed	Blood purifier (4), Gastric discomfort (4)	OTH-A, GAS-D	Burnt ash	Oral	8	B, C
Nitrariaceae	*Peganum harmala* L.	Esfand 1060	Fruit, Seed	Disinfectant (52), Reduce rheumatic pain (2), Reduce blood sugar (9), bruise (18)	OTH-A, SKE-L, MET-T, SKE-L	Poultice, Decoction	Topical, Oral	81	A
Oleaceae	*Jasminum officinale* L.	Yas sefid 1065	Flower	Antivirus (2), Relaxing (6)	OTH-A, NER-N	Infusion	Oral	8	B, C
Oleaceae	*Olea europaea* L.	Zeytoon 1066	Leaf, Fruit	Antihypertensive (21), Bone and joint pains (5), Appetizing (2), Urinary, (5), Hair tonic (3)	CAR-K, DER-S, SKE-L, OTH-A, URO-U	Decoction, Oil, Pickle	Oral, Topical	61	A
			Leaf, Fruit	Antibacterial (4)	OTH-A	Decoction, Poultice	Topical, Oral		
			Oil	Reduce blood sugar (5), Remove bur from skin (16)	MET-T, DER-S	Oil, Poultice	Topical, Oral		
Oleaceae	*Syringa persica* L*.*	Yas 1069	Stem bark, Fruit	Relaxing (3)	NER-N	Infusion	Oral	3	B, C
Onagraceae	*Epilobium angustifolium* L.	Bid alafi 1070	Leaf and seed	Pesticide (4), Anti-inflammation of mucosa and mouth (6), Wound healing (9), Heart tonic (8), Febrifuge (7)	OTH-A, OTH-A, DER-S, CAR-K, OTH-A	Decoction	Oral	34	A
Onagraceae	*Epilobium hirsutum* L.	Bid alafi korkee 1072	Leaf, Root	Relaxing (5)	NER-N	Infusion	Oral	5	B, C
Orobancheaceae	*Orobanche ramosa* L*.*	Poor 1080	Stem	Stomachache (19)	GAS-D	Edible, Decoction	Oral	19	B, C
Papaveraceae	*Fumaria indica* (Hausskn.) Pugsley.	Shatereh 1090	Leaf and young branches	Anti-diarrhea (5), Anti-nausea (4), Stomachache (5)	GAS-D, GAS-D, GAS-D	Decoction	Oral	14	A
Papaveraceae	*Fumaria officinalis* L.	Shatreh 1091	Foliage	Blood purifier (6)	OTH-A	Infusion	Oral	6	A, C
Papaveraceae	*Fumaria parviflora* Lam.	Shatereh 1093	Leaf and young branches	Anti-diarrhea (3), Anti-nausea (4), Stomachache (4), Blood purifier (6), Diuretic (3), Cutaneous itching (5)	GAS-D, GAS-D, GAS-D, OTH-A, URO-U, DER-S	Decoction	Oral	25	A
Papaveraceae	*Fumaria vaillantii* Loisel.	Shatereh1095	Leaf and young branches	Anti-diarrhea (3), Anti-nausea (4), Stomachache (4)	GAS-D, GAS-D, GAS-D	Decoction	Oral	11	B, C
Papaveraceae	*Hypecoum pendulum* L.	Shatereh 1096	Root, Leaf	Cough (3), Anti-diarrhea (2)	RES-R, GAS-D	Decoction	Oral	5	B, C
Papaveraceae	*Papaver dubium* L.	Taryakoo 1098	Bulb, Leaf	Eczema (3), Acne (4), Anti-inflammatory (5), Bronchitis (6), Cough (5), Pain relief (3)	DER-S, DER-S, SKE-L, RES-R, RES-R, NER-N	Mixed with vinegar, Decoction	Topical, Oral	32	B, C
Papaveraceae	*Roemeria hybrida* (L.) DC.	Shagayeg 1099	Flower	Pain relief (9)	NER-N	Decoction	Oral	9	B, C
Pedalicaceae	*Sesamum indicum* L*.*	Konjed 1140	Seed	Prevention of hair loss (24), Blood fat (5)	DER-S, Blood-B	Decoction, Oil	Oral, Topical	29	A
Plantaginaceae	*Plantago amplexicaulis* Cav.	Tangbar 491	Leaf, Seed	Wound healing (5), Allergy (3), Heatstroke (2), Infectious disease (3), Stomachache (2), Respiratory ailments (2)	DER-S, OTH-A, OTH-A, OTH-A, GAS-D, RES-R	Poultice, Decoction	Topical, Oral	17	B, C
Plantaginaceae	*Plantago ciliata* Desf*.*	Kowchak 493	Leaf, Seed	Antibacterial (4), Burns (9), Anti-inflammatory (2), Constipation (3)	OTH-A, DER-S, SKE-L, GAS-D	Poultice, Decoction, Syrup	Topical, Oral	13	A
Plantaginaceae	*Plantago gentianoides* Sm.	Tangbar 494	Leaf, Seed	Constipation (5), Anti-inflammatory (5), Cough (4)	GAS-D, SKE-L, RES-R	Decoction	Oral	14	B, C
Plantaginaceae	*Plantago indica* L.	Kowchak 495	Seed	Anti-inflammatory (2), Constipation (3)	SKE-L, GAS-D	Decoction, Poultice, Syrup	Topical, Oral	5	B, C
Plantaginaceae	*Plantago lanceolata* L.	Kowchak 490	Leaf, Root,Seed	Baby jaundice (22), Constipation (18), Blood coagulation (3), Asthma (4), Stomachache (6)	OTH-A, GAS-D, Blood-B, RES-R, GAS-D	Dressing, Decoction	Topical, Oral	40	A
Plantaginaceae	*Plantago major* L.	Barhang 492	Aerial parts	Dry cough (6), Itchy throat (8), Alzheimer (5), Cancer (2), Anti-inflammatory (3), Baby jaundice (21), Cough (2), Expectorant (4), Burn healing (4)	RES-R, RES-R, NER-N, CAN-C, SKE-L, OTH-A, RES-R, RES-R, DER-S	Dressing, Decoction	Topical, Oral	28	A
Plantaginaceae	*Plantago ovata* Forssk.	Tokhm sefid 499	Seed	Anti-inflammatory (2), Constipation (3)	SKE-L, GAS-D	Decoction, Poultice, Syrup	Topical, Oral	5	B, C
Plantaginaceae	*Veronica anagallis* L*.*	Sizab 489	Aerial parts	Stomach tonic (10), Diuretic (7)	GAS-D, URO-U	Decoction	Oral	17	B
Platanaceae	*Platanus orientalis* L.	Chenar 487	Fruit, Root, Leaf, Stem bark	Acne (3), Snakebite (3), Hoarseness (2)	DER-S, DER-S, OTH-A	Poultice, Decoction	Oral, Topical	8	B, C
Plumbaginaceae	*Acantholimon scorpius* (Jaub.&Spach) Boiss	Kharposhtoo 511	Root	Livestock wound healing (6), Washing powder (10)	DER-S, OTH-A	Poultice, Powder	Topical	16	B, C
Poaceae	*Avena sativa* L.	Jow dosar 334	Leaf, Seed	Disinfectant (3)	OTH-A	Decoction	Topical	3	B, C
Poaceae	*Cymbopogon schoenanthus* (L.) Spreng.	Kaboo 335	Leaf and stem	Body tonic (4)	OTH-A	Decoction	Oral	4	B, C
Poaceae	*Cynodon dactylon* (L.) Pers.	Marg 337	Aerial parts	Anti-diarrhea (3), Asthma (2)	GAS-D**,** RES-R	Decoction	Oral	8	B, C
Poaceae	*Desmostachya bipinnata* (L.) Stapf	Kerteh 339	Root	Body tonic (4)	OTH-A	Decoction	Oral	4	B, C
Poaceae	*Hordeum distichon* L.	Jow 329	Fruit	Febrifuge (2), Reducing thirst (3)	OTH-A, OTH-A	Decoction	Oral	7	B, C
Poaceae	*Hordeum vulgare* L.	Jow 328	Fruit	Reduce blood sugar (3)	MET-T	Decoction,	Oral	7	A
			Buds	Acne (4)	DER-S	Mask	Topical		
Poaceae	*Melica persica* Kunth	Oshlom 325	Aerial parts	Washing powder (7)	OTH-A	Powder	-	7	B, C
Poaceae	*Phragmites australis* (Cav.) Trin. ex Steud.	Ney 324	Root and Rhizome	Breast milk reduction (5)	OTH-A	Pickle	Oral	5	A, C
Poaceae	*Setaria italica* (L.) P. Beauv.	Garch 340	Seed	Hair tonic (5), Carminative (2)	DER-S, GAS-D	Poultice, Decoction	Topical, Oral	7	A
Poaceae	*Sorghum halepense* (L.) Pers.	Garch 342	Seed	Diuretic (6)	URO-U	decoction	Oral	5	A
Poaceae	*Triticum aestivum* L.	Gandom 345	Oil of seed	Eczema (6)	DER-S	Poultice	Topical	6	B, C
Poaceae	*Zea mays* L*.*	Zorat 347	Style	Kidney stone (27)	URO-U	Decoction	Oral	37	A, C
Polygonaceae	*Polygonum persicaria* L*.*	Bandvash 430	Leaf and flower	Asthma (8), Constipation (5)	RES-R, GAS-D	Infusion, Oil, Aromatic water	Oral	13	B, C
Polygonaceae	*Pteropyrum aucheri* Jaub. and Spach	Perent 425	Foliage	Acne (4), Infectious wounds (5)	DER-S, ER-S	Poultice	Topical	9	B, C
Polygonaceae	*Rheum ribes* L.	Rohoo 432	Aerial parts	Reduce blood sugar (5), Stomach and liver tonic (3), Appetizing (2), Laxative (6), Blood purifier (3), Vermicide (2), Bone tonic (4), Sight Enhancement (6)	MET-T, GAS-D, OTH-A, GAS-D, OTH-A, GAS-D, SKE-L, EYE-F	Decoction	Oral	31	B, C
Polygonaceae	*Rumex crispus* L.	Torshak 437	Aerial parts	Laxative (4), Acute pulmonary embolism (7), Detoxificant of body (5)	GAS-D, RES-R, GAS-D	Decoction	Oral	16	B, C
Polygonaceae	*Rumex vesicarius* L.	Torshak 438	Laef and petiole	Reduce blood sugar (2)	GAS-D	Vegetable	Oral	2	A
Portulacaceae	*Portulaca oleracea* L.	Gholfeh 561	Leaf, Seed	Stomach tonic (1), Reducing thirst (2), Febrifuge (1), Cough (6), Blood purifier (12)	GAS-D, -, OTH-A, RES-R, OTH-A	Vegetable	Oral	21	B, C
Primulaceae	*Anagallis arvensis* L*.*	Delpasand 1145	Aerial parts	Liver cysts (4), Urinary stones (4)	GAS-D, URO-U	Infusion	Oral	8	A
Primulaceae	*Lysimachia maritima* (L.) Galasso, Banfi & Soldano.	Shabdari 1147	Whole plant	Antispasmodic (5), Bronchitis (3)	NER-N, RES-R	Decoction	Oral	8	B, C
Primulaceae	*Primula capitellata* Boiss.	Pamchoo1149	Root and flower	Vermicide (4), Antispasmodic (8)	GAS-D, NER-N	Aromatic water	Oral	12	B, C
Primulaceae	*Samolus valerandi* L.	Alaf 1150	Aerial parts	Body tonic (4)	OTH-A	Decoction	Oral	4	A
Pteridaceae	*Adiantum capillus-veneris* L.	Siahlengoo 1170	Leaf	Common cold (49), Expectorant (51), Relaxing (3), Menstrual disorders (2), Earache (3)	RES-R, RES-R, NER-N, GYN-X, Ear-H	Decoction	Oral	110	A, C
Ranunculaceae	*Adonis aestivalis* L*.*	Chashm gargavol 1180	Whole plant	Anti-inflammatory (5)	SKE-L	Decoction	Oral	5	B, C
Ranunculaceae	*Adonis microcarpa* DC.	Chashm gargavo 1182	Flower	Anti-inflammatory (3)	SKE-L	Decoction	Oral	3	B, C
Ranunculaceae	*Anemone biflora* DC.	Shagayeg neman1185	Flower	Common cold (6)	RES-R	Decoction	Oral	6	B, C
Ranunculaceae	*Clematis ispahanica* Boiss*.*	Chaspakoo 1187	Aerial parts	Diuretic (3), Joint pain (3), Headache (3), Eczema and psoriasis (3)	URO-U, SKE-L, NER-N, DER-S	Decoction	Oral	12	A
Ranunculaceae	*Consolida rugulosa* (Boiss.) Schrödinger	Zaban mooshoo 1189	Aerial parts	Anti-inflammatory (4)	SKE-L	Decoction	Oral	4	B, C
Ranunculaceae	*Nigella sativa* L*.*	Siahdaneh 1190	Seed	Blood pressure (7), Blood fat (7), Asthma (2)	CAR-K, Blood-B, RES-R	Infusion	Oral	16	B, C
Ranunculaceae	*Ranunculus arvensis* L.	Alaleh 1192	Flower	Urinary disease (3)	URO-U	Aromatic water	Oral	3	B, C
Ranunculaceae	*Ranunculus muricatus* L.	Alaleh 1193	Root, Leaf, Flower	Skin diseases (2)	DER-S	Poultice	Topical	2	B, C
Ranunculaceae	*Thalictrum minus* L.	Sadabi 1195	Aerial parts	Gastric discomfort (4)	GAS-D	Decoction	Oral	4	A
Resedaceae	*Ochradenus aucheri* Boiss*.*	Kolirim1200	Leaf	Parasite repellent (2), Wound healing (3)	GAS-D	Decoction, Poultice	Oral	5	B, C
Resedaceae	*Reseda aucheri* Boiss*.*	Varas 1205	Leaf	Laxative (1), Diuretic (3) Reducing thirst (3)	GAS-D, URO-U, OTH-A	Decoction, Row	Oral	5	B, C
Rhamnaceae	*Rhamnus persica* Boiss. & Hohen	Titoomari 1210	Fruit	Anti-diarrhea (11)	GAS-D	Decoction	Oral	11	B, C
Rhamnaceae	*Rhamnus prostrata* Jacq*.*	Titoomari 1211	Fruit	Anti-diarrhea (5)	GAS-D	Decoction	Oral	5	B, C
Rhamnaceae	*Sageretia thea* (Osbeck) M.C. Johnst. Ch	Bastel 215	Fruit	Laxative (2), Blood purifier (2)	GAS-D, CAR-K	Decoction	Oral	4	A
Rhamnaceae	*Ziziphus jujuba* Mill.	Annab 1218	Fruit	Bronchitis (3), Common cold (4), Laxative (20)	RES-R, RES-R, GAS-D	Infusion, Edible	Oral	24	B, C
Rhamnaceae	*Ziziphus nummularia* (Burn.f.)Wight & Am.	Konar 1220	Leaf	Common cold (3), Antimicrobial (1)	RES-R, OTH-A	Decoction, Poultice	Oral, Topical	4	A
Rhamnaceae	*Ziziphus spina-christi* (L.) Desf.	Konar 1221	Leaf, Fruit	Stomach tonic (5), Hair tonic (4), Infectious tuber (2), Skin rash (7)	GAS-D, DER-S, DER-S, DER-S	Eat as fruit, Poultice, Shampoo	Oral, Topical	18	A
Rosaceae	*Agrimonia eupatoria* L*.*	Ghafes 370	Aerial parts	Wound healing (18), Fatty liver (8), Anti-diarrhea (7)	DER-S, GAS-D, GAS-D	Poultice, Decoction	Topical, Oral	33	A, C
Rosaceae	*Amygdalus elaeagnifolia* Spach	Arjan 372	Stem, Fruit	Nuts (18), Eczema (7)	OTH-A, DER-S	burning of semi dried wood, Edible	Oral,Topdical	25	B, C
Rosaceae	*Amygdalus wendelboi* Freitag	Archen 373	Latex	Eczema (4), Bone and joint pains (3)	DER-S	Latex of burning stem	Topical	7	B, C
Rosaceae	*Cotoneaster kotschyi* (C.K.Schneid.) G.Klotz	Siahchoo 375	Latex, Leaf	Jaundice (5), Constipation (6), Dry cough(4)	GAS-D, GAS-D, RES-R	Infusion	Oral	15	A
Rosaceae	*Crataegus ambigua* C.A.Mey. ex A.K.Becker	Kalkouhi 365	Leaf, fruit, Flower	Relaxing (5), Spasms (4), Cardiac distress (4), Hypertension (4), Anti-diarrhea (7)	NER-N, NER-N, CAR-K, CAR-K, GAS-D	Decoction, Salad	Oral	24	B, C
Rosaceae	*Crataegus azarolus* L.	KalKoohi 380	Leaf and fruit	Antihypertensive (6), Relaxing (11), Antispasmodic (10), Cardiac distress (5)	CAR-K, NER-N, NER-N, CAR-K	Decoction	Oral	32	A
Rosaceae	*Crataegus meyeri* Pojark.	Kalkouhi 381	Leaf, fruit, Flower	Relaxing (5), Spasms (4), Cardiac distress (4), Antihypertensive (4)		Decoction	Oral	17	B, C
Rosaceae	*Cydonia oblonga* Mill.	Beh 351	Seed	Sore throat (4)	OTH-A	Decoction	Oral	4	B, C
Rosaceae	*Prunus dulcis* (Mill.) D.A.Webb	Badam-e shirin 353	Seed	Hair tonic (5), Preventing of hair loss (9)	DER-S, DER-S	Oil	Topical	14	B, C
Rosaceae	*Prunus eburnea* (Spach) Aitch. & Hemsl.	Archen 354	Fruit, Gum	Bone and joint pains (6), Allergies (5), Hair tonic (3)	OTH-A, DER-S	Decoction, Poultice	Oral, Topical	14	B, C
Rosaceae	*Prunus lycioides* (Spach) C.K.Schneid.	Badame koohi 355	Leaf and fruit	Preventing of hair loss (5)	DER-S	Poultice, Oil	Topical	8	B, C
Rosaceae	*Prunus mahaleb* L.	Mahlab 356	Leaf bark	Relaxing (5), Liver cysts (4), Parasite repellent (4), Joint pain (4)	NER-N, GAS-D, GAS-D, SKE-L	Decoction, dressing	Oral, Topical	17	A
Rosaceae	*Prunus orientalis* (Mill.) Koehne	Archen 357	Fruit	Bone and joint pains (6), Allergies (5), hair tonic (3)	OTH-A, DER-S	Decoction, Poultice	Oral, Topical	14	B, C
Rosaceae	*Prunus persica* (L.) Batsch	Holo 358	Fruit	Laxative (6)	GAS-D	Nuts, Maceration	Oral	6	B, C
Rosaceae	*Prunus scoparia* (Spach) C.K.Schneid.	Badam-e koohi 376	Seed	Anti-dandruff (29), Preventing of hair loss (27), Earache (3), Health and beauty of the skin (8), Cancer prevention (3), Burned wound healing (4)	DER-S, DER-S, Ear-H, DER-S, CAN-C, DER-S	Poultice, Nut, Oil	Topical, Oral	74	B
			Stem bark	Stomach tonic (4)	GAS-D	Decoction	Oral		
Rosaceae	*Prunus avium* (L.) L*.*	Gilas 378	Pedicel	Kidney stone (7)	URO-U	Decoction	Oral	24	A, C
Rosaceae	*Prunus cerasus* L.	Albaloo 359	Pedicel	Kidney stone (5)	URO-U	Decoction	Oral	5	A, C
Rosaceae	*Rosa beggeriana* Schrenk ex Fisch. & C.A.Mey.	Roz sefid 360	Flower	Stomach tonic (5), Relaxing (8)	GAS-D, NER-N	Decoction	Oral	13	B, C
Rosaceae	*Rosa canina* L*.*	Korrik 362	Leaf, Flower, Fruit	Relaxing (5)	NER-N	Decoction	Oral	5	B, C
Rosaceae	*Rosa damascena* Herrm*.*	Gole mohammadi 374	Flower	Flavoring of food (8), Laxative (25), Nervous tonic (43)	OTH-A, GAS-D, NER-N	Aromatic water	Oral	76	B, C
Rosaceae	*Rosa moschata* Herrm*.*	Korrik 377	Flower	Nervous tonic (3)	NER-N	Decoction	Oral	3	B, C
Rosaceae	*Rubus caesius* L*.*	Saghder 371	Leaf, Fruit	Laxative (14)	GAS-D	Decoction, Syrup	Oral	14	A
Rosaceae	*Sanguisorba minor* Scop.	Gheytaran 379	Fruit	Common cold (3), Relaxing (4), Cough (4), Jaundice (5), Toothache (6)	RES-R, NER-N, NER-N, GAS-D, GAS-D	Decoction	Oral	32	A
Rubiaceae	*Plocama aucheri* (Guill.) M.Backlund & Thulin	Khargo l520	Foliage	Bone and joint pain (3), Reduce rheumatic pain (2), Reduce blood sugar (3), Digestive (3)	SKE-L, SKE-L, MET-T, GAS-D	Decoction, Infusion, Poultice	Topical, Oral	11	B, C
Rubiaceae	*Rubia albicaulis* Boiss.	Roonask 523	Fruit	Bone tonic (4), Constipation (3)	SKE-L, GAS-D	Decoction, Infusion	Oral	7	B, C
Rubiaceae	*Rubia tinctorum* L*.*	Roonask 524	Root	Bone tonic (4), Constipation (3)	SKE-L, GAS-D	Decoction, Infusion	Oral	7	B, C
Rutaceae	*Citrus aurantium* L.	Nareng 527	Flower	Anti-diarrhea (5), Relaxing (8), Eye diseases (8), Traditional kohl (3)	GAS-D, NER-N, EYE-F	Decoction, Kohl	Oral	24	B, C
Rutaceae	*Citrus limon* (L.) Osbeck	Limoo torsh 528	Fruit, Seed	Eye diseases (15), Traditional kohl (3)	EYE-F	Juice, Kohl	Eye Drop	18	B, C
Rutaceae	*Haplophyllum robustum* Bunge	Sadoo 530	Aerial parts	Gastric discomfort (1)	GAS-D	Decoction	Oral	1	B, C
Rutaceae	*Haplophyllum tuberculatum* Juss.	Gahich 531	Aerial parts	Febrifuge (2), Headache (2)	GAS-D, NER-N	Decoction, Poultice	Oral, Topical	4	A
Rutaceae	*Ruta graveolens* L*.*	Soddab 535	Leaf	Urinary stone (6)	URO-U	Decoction	Oral	6	A
Salicaceae	*Populus alba* L*.*	Sepidar 600	Stem bark and leaf	Blood purifier (4), Pain relief (3)	OTH-A, NER-N	Decoction	Oral	7	A
Salicaceae	*Populus euphratica* Oliv*.*	Senewbar 601	Stem bark	Parasite repellent (7)	GAS-D	Decoction		4	A
Salicaceae	Salix aegyptiaca L*.*	Beedmeshk 605	Inflorescence	Laxative (3), Anti-diarrhea (2), Gastrointestinal pains (2), Menstruation pains (3)	GAS-D, GAS-D, GAS-D, GYN-X	Decoction, Aromatic water	Oral	10	A
Salicaceae	*Salix alba* L.	Beed 606	Stem bark, Leaf	Febrifuge (21), Common cold (6)	OTH-A, RES-R	Decoction, Aromatic water	Oral	27	A
Salvadoraceae	*Salvadora oleoides* Decne*.*	Pir 1230	Fruit	Appetizing (7), Laxative (4), Parasite repellent (10), Hemorrhoids (4), Bronchitis (3)	OTH-A, GAS-D, GAS-D, CAR-K, RES-R	Decoction, Edible	Oral	28	B, C
Salvadoraceae	*Salvadora persica* L.	Chooch1231	Fruit	Appetizing (5), Expectorant (8)	OTH-A, RES-R	Decoction, Edible	Oral	15	B, C
Scrophulariaceae	*Scrophularia scopolii* Hoppe ex Pers.	Makhleseh 842	Young stem, Fruit	Waist pain (3), Respiratory diseases (2)	OTH-A, SKE-L	Poultice, Decoction	Topical, Oral	5	A, C
Scrophulariaceae	*Scrophularia striata* Boiss.	Makhleseh 840	Fruit	Gastric discomfort (2), Respiratory diseases (2), Waist pain (3), Wound healing (2)	GAS-D, RES-R, OTH-A, DER-S	Poultice, Decoction	Oral, Topical	9	A, C
Solanaceae	*Datura stramonium* L.	Tatooreh 1250	Leaf, Seed	Sexual tonic (6), Bone and join pains (8), Reduce rheumatic pain (5), Asthma (5), Cough (4), Burn (5)	OTH-A, SKE-L, SKE-L, RES-R, RES-R, DER-S	Decoction, Poultice	Oral, Topical	33	B, C
Solanaceae	*Hyoscyamus reticulatus* L.	Bonji 1255	Aerial parts	Pain relief (9), Leaving addiction (9), Narcotic (15)	NER-N, NER-N, RES-R	Infusion	Oral	33	A, C
Solanaceae	*Hyoscyamus senecionis* Willd.	Bangdaneh 1256	Flower	Pain relief (8)	NER-N	Decoction	Oral	8	A, C
Solanaceae	*Hyoscyamus squarrosus* Griff.	Bangdaneh 1257	Whole plant	Pain relief (8)	NER-N	Decoction	Oral	8	A, C
Solanaceae	*Lycium barbarum* L.	Zeel 1260	Fruit	Sleeplessness (4)	NER-N	Eat as fruit	Oral	8	B, C
Solanaceae	*Lycium depressum* Stocks	Zeel 1261	Fruit	Anticonvulsant (11)	OTH-A, NER-N	Decoction	Oral	11	B
Solanaceae	*Lycium shawii* Roem. & Schult.	Dahir1262	Leaf	Vision enhancement (2)	EYE-F	Crashed Juice	Topical	2	B, C
Solanaceae	*Physalis alkekengi* L.	Aroosak-e posht-e pardeh 1265	Fruit	Kidney diseases (3), Laxative (5), Expectorant (2)	OTH-A, GAS-D, RES-R	Edible	Oral	10	B, C
Solanaceae	*Solanum nigrum* var.villosum L.	Roopas 1268	Fruit	Febrifuge (6), Blood coagulation (5), Pain relief (4)	OTH-A, Blood-B, NER-N	Decoction	Oral	15	A
Solanaceae	*Solanum lycopersicum* L.	Gewjeh 1251	Fruit	Infectious wounds (5)	DER-S	Poultice	Topical	5	B, C
Solanaceae	*Withania somnifera* (L.) Dunal	Kahkenj 1270	Aerial parts	Nerve tonic (5)	NER-N	Decoction	Oral	5	A
Tamaricaceae	*Tamarix aphylla* (L.) Karst.	Koor gaz 1350	Latex of burning stem	Eczema (16), Skin disease (8)	DER-S, DER-S	Poultice	Topical	24	B, C
Tamaricaceae	*Tamarix kotschyi* Bunge	Gole kist 1351	Latex of burning stem	Eczema (16), Skin disease (8)	DER-S, DER-S	Poultice	Topical	24	B, C
1Thymelaeaceae	*Daphne mucronata* Royale	Terveet 704	Leaf	Influenza (3), Arthritis (3), Blood cancer (1)	MET-T, RES-R, SKE-L, CAN-C	Decoction, Dressing	Oral, Topical	7	A
Thymelaeaceae	*Daphne oleoides* Schreb.	Terveet 705	Foliage	Traditional dyeing (10), Natural color for textile (10)	OTH-A, OTH-A	Decoction, Fume	-	24	B, C
			Foliage	Constipation (4)	GAS-D	Decoction	Oral		
Thymelaeaceae	*Daphne stapfii* Bornm.& Keisslere.	Terveet 706	Fruit	Influenza (3), Arthritis (3)	MET-T, RES-R, SKE-L	Decoction, Fume, Poultice	Oral, Inhale, Topical	6	B, C
Thymelaeaceae	*Diarthron lessertii* (Wikstr.) Kit Tan	Gole bidi 708	Leaf and flowering branches	Stomachache (2), Stomach and liver tonic (2), Arthritis (2)	GAS-D, GAS-D, SKE-L	Aromatic water, Poultice	Oral, Topical	6	B, C
Urticaceae	*Parietaria judaica* L.	Gooshe Moosh 323	Leaf and flower	Diuretic (3), Acute pulmonary embolism (4)	URO-U, RES-R	Decoction	Oral	7	B, C
Urticaceae	*Urtica dioica* L.	Soosonakoo 320	Leaf and flower	Urinary stone (7), Reduce blood sugar (4)	URO-U, MET-T	Infusion	Oral	11	A
Urticaceae	*Urtica urens* L.	Soosonakoo 321	Aerial parts	Febrifuge (5), Gastrointestinal pains (4), Relaxing (2), Anti parasite (2), Toothache(3)	OTH-A, GAS-D, NER-N, GAS-D, GAS-D	Decoction, Aromatic water	Oral	15	B, C
Violaceae	*Viola odorata* L.	Gol-e banafsheh 1370	Leaf and flower	Laxative (8), Chronic cough (5), Expectorant (3)	GAS-D, RES-R, RES-R	Decoction	Oral	16	B, C
Verbenaceae	*Verbena officinalis* L.	Shahpasand 1380	Aerial parts	Febrifuge (3), Nerve tonic (7)	OTH-A, NER-N	Poultice, Decoction	Topical	10	A
Vitaceae	*Vitis vinifera* L.	Maviz 1390	Dried Fruit	Memory improvement (5)	NER-N	Nuts	Oral	5	A
Xanthorrhoeaceae	*Aloe vera* (L.) Burm.f.	Alovera 1500	glazed materials	Diabetic wound (7), Eczema (22)	DER-S, DER-S	Poultice	Topical	29	A
Xanthorrhoeaceae	*Asphodelus tenuifolius* Cav.	Peemazoo1505	Seed	Diuretic (15)	URO-U	Decoction	Oral	17	A
			Root	Herbal adhesive (2)	OTH-A	Crushed extract	-		
Xanthorrhoeaceae	*Eremurus kopetdaghensis* M.Pop.ex B.Fedtsch.	Horishoo 1510	Root, Leaf and flower	Herbal adhesive (4), Vegetable (8), Jaundice (6), Liver Disease (5), Disinfectant (4)	OTH-A, OTH-A, GAS-D, GAS-D, OTH-A	Vegetable, Decoction. Poultice	Topical, Oral	27	B, C
Xanthorrhoeaceae	*Eremurus persicus* (Jaub. & Spach) Boiss.	Serishoo 1511	Leaf and flower	Flavoring of food (15), Laxative (6), Herbal adhesive (4), Vegetable (7), Disinfectant (3), Jaundice (6), Liver and kidney Disease (2)	OTH-A, GAS-D, OTH-A, OTH-A, OTH-A, GAS-D, GAS-D	Powder, Vegetable, Decoction. Poultice	Topical, Oral	52	B, C
			Root	Liver and stomach discomfort (31)	GAS-D	Powder	Oral		
Zygophyllaceae	*Fagonia bruguieri* DC.	Alaf kharoo 1550	Aerial parts	Appetizing (2), Vermicide (2), Carminative (5)	OTH-A, GAS-D, GAS-D	Decoction, infusion	Oral	9	A
Zygophyllaceae	*Tribulus terrestris* L.	Kharkhesak 1555	Aerial parts	Kidney stone (43)	URO-U	Decoction	Oral	43	A
Zygophyllaceae	*Zygophyllum eurypterum* Boiss. & Buhse.	Gich 1560	Seed	Lactiferous (4), Anti-nausea (3), Stomach tonic (4), Laxative (4), Vermicide (3)	PRE-W, GAS-D, GAS-D, GAS-D, GAS-D	Decoction	Oral	18	B, C
*Zygophyllaceae*	*Zygophyllum fabago* L.	Gich 1561	Seed	Lactiferous (4), Anti-nausea (3), Stomach tonic (4), Laxative (4), Vermicide (3)	PRE-W, GAS-D, GAS-D, GAS-D, GAS-D	Decoction	Oral	18	B, C

C: indicate the medicinal plants which reported in this region for the first. A: indicate the ethno-medicinal uses of the medicinal plants which quoted in the in the Persian ethnobotany, B: was not quoted in the in the Persian ethnobotany.

### Ailment categories

All recorded ailments and medicinal plant uses were categorized based on the International Classification of Primary Care (ICPC-2) (http://www.who.int/classifications/icd/adaptations/icpc2/en/).

Some modifications were made for diseases such as low back pain, which were not matched with the broad classification of diseases. Therefore, low back pain, infections, and some nontherapeutic applications (pickle, flavoring of food, appetizing, vegetable, thirst, pest control, food coloring, herbal adhesive, and washing powder) were placed in the General and Unspecified category.

According to the results, 16 disease categories were set, namely: (1) General and Unspecified; (2) Gastrointestinal; (3) Ophthalmological; (4) Ear, Nose and Throat; (5) Cardiovascular; (6) Hematological and immune mechanism; (7) Musculoskeletal; (8) Neurological; (9) Psychological; (10) Respiratory; (11) Dermatological; (12) Endocrine/ Metabolic and Nutritional; (13) Urological; (14) Pregnancy, Childbearing, Family Planning; (15) Female Genitals; and (16) Cancer.

### Data analysis

Data was analyzed using descriptive and quantitative statistical methods. In this regard, the ethnomedicinal data was analyzed using frequency, citation, and use reports. Use report was recorded whenever an informant cited a plant species or part(s) used for a particular ailment. Use reports were also quantified to define the highly used plant species for a particular ailment. Additionally, ICF was employed to determine the homogeneity of the information as follows:

ICF = Nur − Nt / Nur − 1

Nur is the number of use citations for each ailment category and Nt is the number of plant species used for the same ailment category by all the healers [20]. ICF ranged between 0 and 1. ICF value is higher (near to 1) when a few plant species are cited by a higher proportion of healers, indicating homogeneity of information about the usage of specific plants. A low value (close to 0) demonstrates the healers’ disagreement about the usage of the plant for a particular ailment category [21].

In order to find out the importance of a specific plant species by informants, the index of relative frequency of citation (RFC) was calculated by dividing the frequency of citation (FC) by the total frequency of informants (RFC = FC/*N*). In this formula, FC is the number of informants who mentioned plant species as useful and *N* is the total frequency of informants in the survey [22].

Moreover, in order to determine cultural significance of each plant species, cultural importance index (CI) [22] was calculated as follows:

$$ \mathrm{CI}=\sum \limits_{u={u}_1}^{u_{\mathrm{nc}}}.\sum \limits_{i={i}_1}^{i_n}\ {\mathrm{UR}}_{\frac{ui}{N}} $$

Independent samples *t*-test was run to compare medicinal uses between men and women. One-way ANOVA and post hoc was used to compare medicinal uses among age groups, educational levels, and occupations.

## Results and discussion

### Botanical diversity

In this ethnobotanical survey which covered the whole Kerman province (23 cities and 3,164,716 population), a total of 217 local informant interviews revealed the application of 402 medicinal plants for the treatment of 95 diseases across 16 ICPC ailments categories. These results showed that herbal medicines are mainly used to treat ailments among the local communities and indicated the rich floral diversity of this region.

These species belong to 273 genera of 73 families where 367 species are Dicotyledons, 27 species Monocotyledons, 7 species Cryptogam, and one species Gymnosperm. An important implication of the current study is the identification of the traditional medicinal uses of 292 plant species in this region for the first time. Information about these recorded medicinal plants is summarized in Table 3 in terms of local names, voucher specimens, part(s) used, healing practices, drug preparation, ICPC classification, and use report (%).

**Fig. 2:**
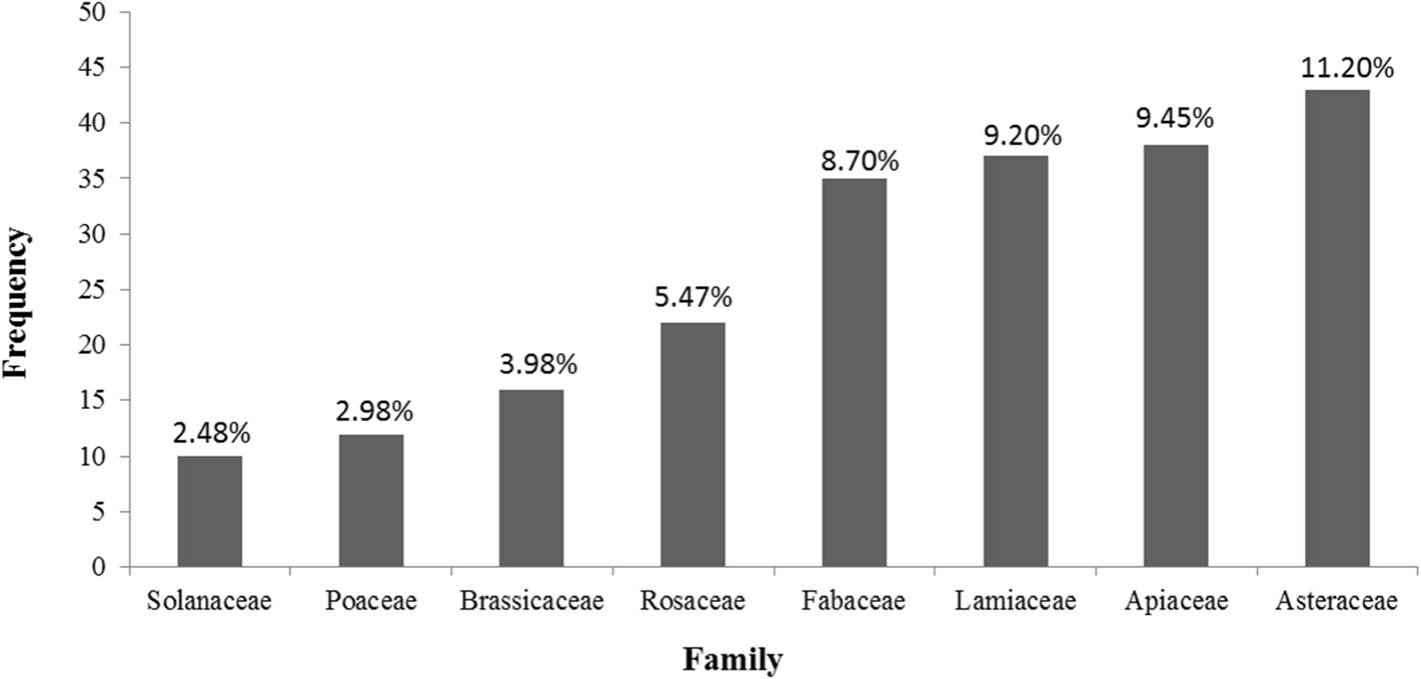
Top dominants medicinally utilized plant families

Asteraceae, Apiaceae, and Lamiaceae with 43, 38, and 37 species were the dominant medicinally utilized plant families, respectively (Fig. 2). In the south of this province, Sadat-Hosseini et al. reported that Apiaceae, Asteraceae, and Lamiaceae are the dominant medicinal plant families [10]. Moreover, in several ethnobotanical studies in the neighboring provinces (in Sistan and Baluchesta [23], and in Isfahan [24]) and countries (like Turkey [25, 26], and Georgia [27]), similar results were reported on the dominance of two or three of these plant families. From the phytochemical point of view, the dominance of Apiaceae, Asteraceae, and Lamiaceae families might be due to phytochemical composition, which are clues to high content of essential oils and phenolic constituents responsible for antimicrobial and antioxidant properties [28, 29].

*Nepeta*, *Prunus*, *Ferula*, *Plantago*, *Ephedra*, *Euphorbia*, *Artemisia*, *Salvia*, *Artemisia*, and *Astragalus* were the dominant medicinally used plant genera. Moreover, the findings of Saber et al. revealed that *Salvia*, *Nepeta*, *Artemisia*, *Astragalus*, *Ferula*, *Plantago*, *Ephedra*, *Amygdalus*, and *Crataegus* are the most frequently and popularly used medicinal plant genera in this district [8]. In general, the therapeutic significance of some plant families in a specific district may be related to the common distribution of their species [30].

There were some species like *Tecomella undulata* that were classified under vulnerable and endangered category of the IUCN list due to low reproduction and overexploitation [31, 32] while *Pergularia tomentosa* is a rare and endangered plant species which grows in the south of Kerman. Our previous research revealed that a low percentage of Kerman rangelands is vegetated with this plant and inhabitants uprooted it to meet their pharmacological needs [33].

The finding showed that the majority of the medicinal plant species (95%) belong to the wild habitat and the rest to cultivated areas. Other reports in this district [16, 27] confirm our results. In this case, informants of this region believed that wild plants are more medicinally effective than cultivated ones. Moreover, similar results were reported by Hu et al. in China [2].

With respect to healthcare policies, despite the relative adequate health services in this study area, local people and herbalists preferred herbal medicine due to the synthetic drugs side effects compared to herbal medicine. Furthermore, the general health policies that have been approved by the fifth development plan, as well as the national document on medicinal plants and traditional medicine, which emphasizes the organization and development of natural and traditional products, could play an important role in shaping people’s inclination towards traditional medicine [34]. Moreover, the medicinal plants’ availability, low cost, positive experiences, and reliable Iranian references like Avicenna could be the other reasons to form positive attitudes. Kerman province is a pivotal state in the ancient Iran (Persia) and it is estimated that the human civilization emerged from Jiroft in the south of this province [11]. Therefore, with a rich history, it has developed a sound traditional health care system.

### Plant parts used

**Fig. 3:**
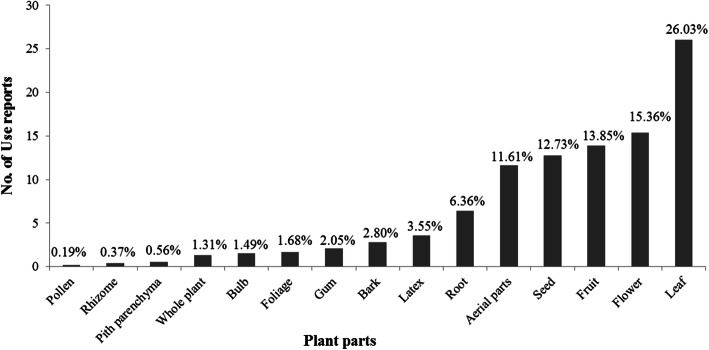
Plant parts used in traditional herbal drug preparation and the number of use reports

To prepare crude drugs (Fig. 3) from 15 plant parts, the most common plant parts used were leaf, flower, fruit, and seeds with 26.03, 15.36, 13.85, and 12.73 percentages, respectively. According to many reports, the leaf is the most common medicinal plant part used in the ethnopharmacological applications [35, 36]. Field discussion and other similar reports [30] indicated that availability, abundance, efficiency of use, and attention to the conservation points are the main reasons for the maximum usage of the leaves by local healers. In fact, local informants believed that different parts of the medicinal plants could have different therapeutic effects. In other words, plant organs have received varying degrees of attention based on traditional herbal medicine experiences of the ethnic communities. For instance, the root of *Berberis integerrima* is decocted and taken orally to treat diabetes while its leaf is used to treat hypertension. In addition, latex of *Calotropis procera* was used to cure eczema but its leaf is taken in the form of poultice to treat bruise and diabetes.

### Preparation and application modes

**Fig. 4:**
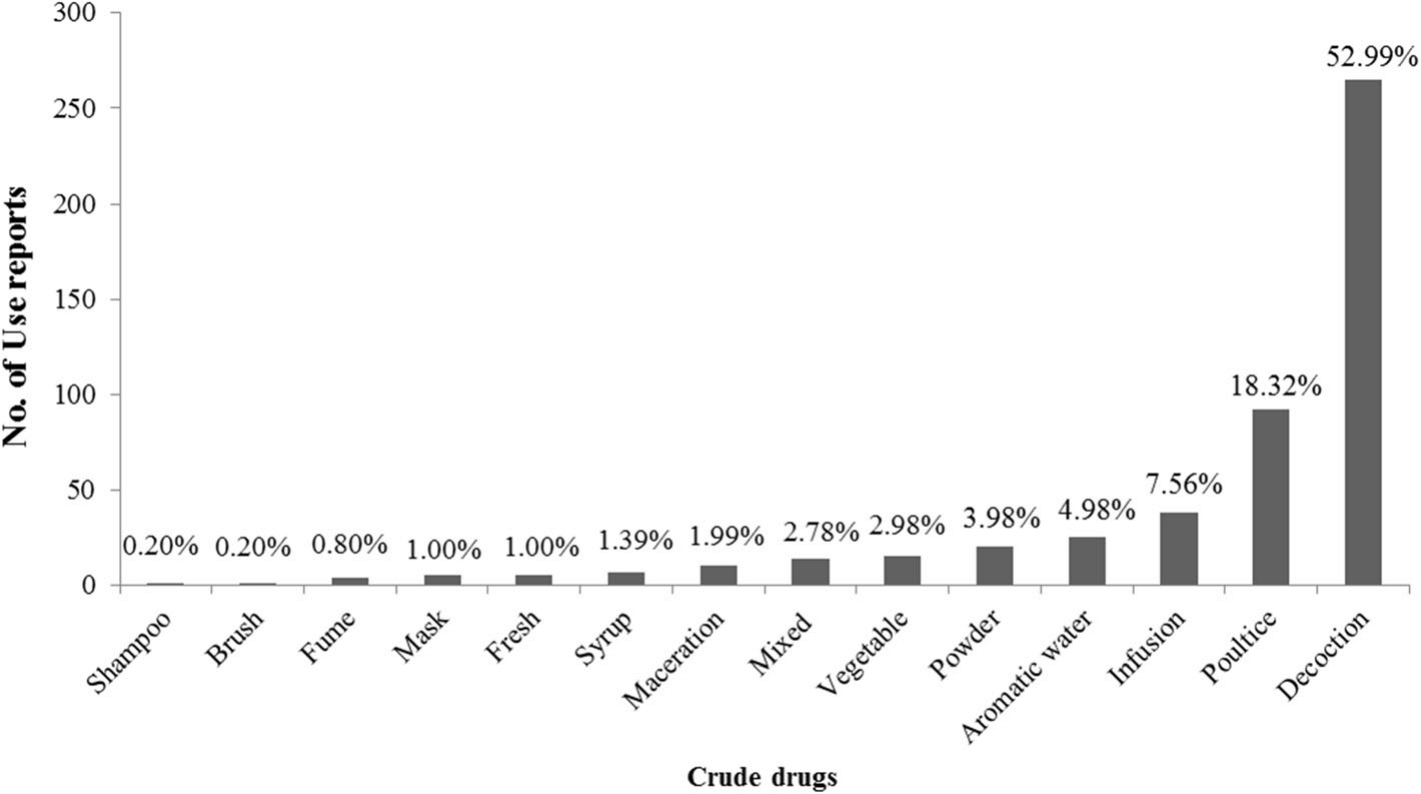
Crude drug type and the number use reports

The medicinal herbs were prepared in 14 forms including decoction, poultice, infusion, aromatic water, powder, vegetable, maceration, syrup, mask, fume, brush, and shampoo by local communities. The most common form of the crude drug was decoction (52.99%), followed by poultice (18.32%) and infusion (7.56%) (Fig. 4). The local informants of Kerman province believed that by decocting the medicinal plants parts, their extract becomes more concentrated and obtains better taste and stronger efficacy. Based on various reports [9, 16, 37–40], decoction is the most common method to prepare herbal medicine.

**Fig. 5:**
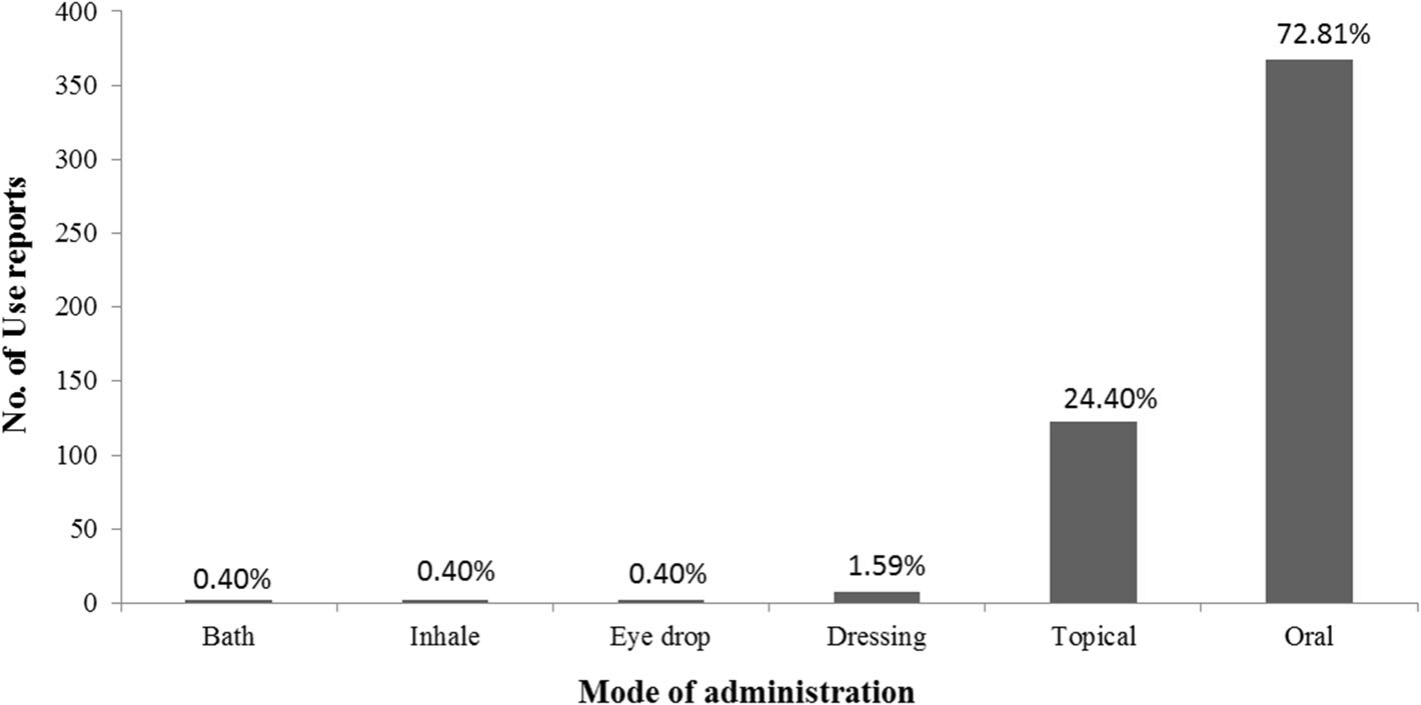
Mode of herbal drug administration and the number of use reports

The medicinal drugs administrated in six categories included oral, topical, dressing, eye drop, inhale, and bath. Analyzing the ethnobotanical data showed that the most common administration route was oral, followed by topical (Fig. 5). Other ethnobotanical studies in Iran and other countries revealed that ethnic communities mostly prefer these two methods of preparation [5, 10, 41]. But some plant species such as *Lawsonia inermis*, *Juniperus excels*, *Rhazya stricta*, and *Pistacia atlantica* are utilized in both topical and oral administration routes. For example, bath with the aqueous extract of *Lawsonia inermis* leaves is known as an effective method for the treatment of jaundice. The poultice of this plant is used to cure skin disorders like eczema and wound scar while its root is decocted and used orally as a diuretic and for the treatment of bronchitis.

### Informant consensus factors (ICF)

The ICF values for different ailment categories treated by the local informants in this survey ranged from 0.25 to 0.92 (Table 4). Endocrine (diabetes), dermatology, gastrointestinal, and respiratory with 0.92, 0.91, 0.90, and 0.89 ICF, respectively, were ranked as the most popular ailment categories for medicinal plants in this region.

**Table 4:** Informant consensus agreement for ailment categories in the Kerman province

ICPC categories	Recorded ailments	Nt*	Nur**	ICF value***
General and Unspecified (OTH-A)	Health and body tonic (88), Fever (279), Disinfectant (116), Detoxification (51), Sore throat (142), Allergy (13), Back pain (6), Undefined illness (59), No diseases (344)	136	1052	0.87
Digestive (GAS-D)	Constipation (146), Toothache (127), Gastritis (395), Intestinal worm (69), Diarrhea (492), Jaundice (191), Nausea (54), Stomachic (781), Tooth germ (2), Liver ailment (115), Carminative (229), Vomiting (16), Digestive (32)	243	2609	0.90
Ophthalmological (EYE-F)	Eye Sight enhancement (5), Pterygium (2), Eye diseases (8)	6	15	0.64
Ear (Ear-H)	Earache (9), Ear diseases (5)	5	14	0.69
Cardiovascular (CAR-K)	Blood pressure (105), Hemorrhoids (13), Heart tonic (13), Cardiovascular disease (6), Blood purifier (115)	37	252	0.85
Blood, Blood Forming Organs and Immune Mechanism (Blood-B)	Anemia (42), Blood coagulation (14), Blood fat (31)	87	16	0.82
Musculoskeletal (SKE-L)	Bone and Joint pains (215), Anti-inflammation (74), Muscular cramps (2), Rheumatism (58), Arthritis (9), Waist pain (6)	60	364	0.83
Neurological (NER-N)	Dizziness (9), Nervous problems (115), Migraine (7), Antispasmodic (93), Pain relief (161), Relaxing (246), Sleeplessness (33) Alzheimer (5), Memory Improvement (18), Depression (5), Headache (40), Leaving addiction (100), Anticonvulsant (7)	112	852	0.86
Psychological (Psy-P)	Anorexia (10)	4	10	0.66
Respiratory (RES-R)	Cough (445), Asthma (37), Respiratory diseases (112), Colds (454), Bronchitis (48), Itchy throat (8), Acute pulmonary embolism (18), Influenza (6)	121	1128	0.89
Skin (DER-S)	Bites (32), Bruise (138), Burn (144), Wound (317), Eczema (368), Skin ailments (105), Skin rash (50), Beauty of skin and hair (13), Acne (22), Scar (61), Blister (5), Skin patches (2), Skin bur, (22) Hair tonic (208), Dandruff (29), Hair leprosy (36), Warts (11)	132	1563	0.91
Endocrine/ Metabolic and Nutritional (MET-T)	Diabetes (310)	25	310	0.92
Urological (URO-U)	Urinary problems (75), Kidney stone (77), Kidney diseases (14)	32	191	0.83
Pregnancy, Childbearing, Family Planning (PRE-W)	Male infertility (11), Lactiferous (13)	6	24	0.78
Female Genital (GYN-X)	Menstrual irregular (79), Women infection (31), Ovarian augmentation (3)	113	16	0.86
Cancer (CAN-C)	Tumorous cancer (9), Blood cancer (1)	7	9	0.25

Nt= number of plant species used in each ailment category; NUR= number of mentions in each used category; ICF= Informant Consensus actor.

Diabetes disorder scored the highest ICF (0.92). This unexpected result is due to the high use report of few medicinal plants for treating diabetes mellitus such as *Citrullus colocynthis*, *Berberis integerrima*, and *Tecomella undulata* with 91, 61, and 41 use reports, respectively*.* In this case, *C. colocynthis*, a reputed medicinal plant in Kerman province, is well known due to its antidiabetic properties [42]. Moreover, in this case, the locals extremely used the root of *Berberis integerrima* and stem bark of *T. undulata* in treating diabetes mellitus. Field surveys and discussion with herbalists revealed that stress caused by poor economic conditions and job pressures can be one of the reasons for the high prevalence of diabetes in this region, but it needs more investigation.

Second ICF was found in dermatological ailment category with 1563 use reports. These findings are due to high use reports for plant species such as *Calotropis procera*, *Pergularia tomentosa*, *Rhazya srticta*, and *Tecomella undulata* in the treatment of eczema, wound healing, and other skin disorders. High ICF of dermatological disorders in this region might be due to high and long-term sunlight exposure, which caused skin disorders [10]. In addition, based on the field surveys, some environmental problems such as dust and wind associated with the particles of sands especially in the cities of Shahdad, Roodbar, Bam, Baravat, Qale Ganj, and Fahraj can be considered critical risk factors. Heidari et al. reported that the main reason for the skin diseases in the desert areas of the Kerman province like city of Bam is the dusty winds [43].

The third highest ICF (0.90) was found in gastrointestinal ailment category for 243 medicinal plant species. Species like *Artemisa* spp. *Glycyrrhiza glabra* and *Nepeta cataria* were typical medicinal herbs for gastrointestinal disorders. Such findings indicated the rich and high level of informant consensus on the variety of medicinal herbs used to treat gastrointestinal ailments, and confirmed the prevalence of gastrointestinal ailments among people who lived in a specific region [30]. Moreover, ITM (e.g., Canon) has attracted considerable attention for the treatment of gastrointestinal disorders [44, 45]. Several studies in Iran and other countries reported that the species *Nepeta cataria* [46, 47], *Glycyrrhiza glabra* [48, 49], and *Artemisa* spp. [50, 51] were traditionally used to treat gastrointestinal diseases.

The 4th disease category with ICF value of 0.89 was respiratory disorders. *Zataria multiflora*, *Thymus fedtschenkoi*, and *Cionura erecta* were major plant species for cough with 104, 102, and 87 use reports, respectively. The large number of use reports for respiratory disorder category might be attributed to undesirable working conditions of local populations, like agriculture and husbandry in the dry and dusty regions without quick access to the health care systems. In confirmation of the present findings, Khanjani et al. studied the relationship between air pollution and respiratory diseases in Kerman from 2006 to 2010 and reported that sandstorms and the dust content increase of the atmosphere exacerbate respiratory diseases in this region [52].

### Use report

Medicinal plants of the families Lamiaceae (such as *Nepeta cataria* and *Zataria multiflora*), Asteraceae (like *Artemisia persica* and *Launaea acanthodes*), and Apiaceae (such as *Bunium persicum*) had the largest number of use report in this area. Bibak and Moghbeli, and Sadat-Hosseini et al. studied the medicinal plants of the Jiroft and Kanuj in the south of Kerman and, similar to the findings of the current study, confirmed the importance of traditional medication of these three families [10, 16]. In addition, plant species of the Apocynaceae family (like *Cionura erecta*, *Rhazya stricta*, and *Calotropis procera*) were ranked with a high use report. In this case, *Cionura erecta* is a well-known medicinal plant in the southern regions of Kerman for the treatment of sore throat and cough with no official records.

### Cultural importance and relative frequency of citation index

The highest CI was found for *Nepeta cataria*, *Zataria multiflora*, *Teucrium polium*, *Rydingia persica*, and *Thymus fedtschenkoi.* The second highest CI was found for *Dracocephalum polychaetum* and *Pistacia atlantica.* These findings revealed that the first CI of the medicinal plants in Kerman province belonged to the Lamiaceae family. Additionally, *Pistacia atlantica* was ranked as an important medicinal plant with high CI index in this region. High CI values show that these medicinal plants are either highly utilized, or their uses are rising in traditional herbal medicine in a specific region [53].

However, for the RFC index, *Lawsonia inermis*, *Artemisia persica*, *Zataria multiflora*, and *Nepeta cataria* were classified as the first rank. In other words, the mentioned medicinal plants were referred by most of the informants. RFC value specifies the usefulness of medicinal plant species [5]. Table 5 shows the ranking based on each index (CI and RFC) for 20 dominant medicinal plant species with the highest CI and RFC indices.

**Table 5:** Comparison of dominant plants by using indices and species ranking based on each index (RFC and CI)

Family	Scientific name	RFC	CI	RFC ranking	CI ranking
Cucurbitaceae	*Citrullus colocynthis* (L) Schrad.	0.42	0.55	4	4
Asteraceae	*Launaea acanthodes* (Boiss.) Kuntze	0.37	0.58	4	4
Fabaceae	*Glycyrrhiza glabra* L.	0.56	0.64	3	4
Cupressaceae	*Juniperus excelsa* M.Bieb.	0.41	0.66	4	4
Apocynaceae	*Calotropis procera* (Aiton) Dryand.	0.63	0.70	2	4
Lamiaceae	*Mentha longifolia* (L.) L.	0.49	0.74	3	3
Lythraceae	*Lawsonia inermis* L.	0.77	0.76	1	3
Apocynaceae	*Cionura erecta* (L.) Griseb.	0.31	0.77	4	3
Apocynaceae	*Rhazya stricta* Decne.	0.63	0.80	2	3
Asteraceae	*Artemisia persica* Boiss.	0.79	0.82	1	3
Berberidaceae	*Berberis integerrima* Bunge	0.66	0.84	2	3
Apiaceae	*Bunium persicum* (Boiss.) B.Fedtsch.	0.73	0.85	2	3
Bignoniaceae	*Tecomella undulata* (Sm.) Seem.	0.51	0.89	3	3
Anacardiaceae	*Pistacia atlantica* Desf.	0.75	0.96	2	2
Lamiaceae	*Dracocephalum polychaetum* Bornm.	0.64	1.00	2	2
Lamiaceae	*Thymus fedtschenkoi* Ronneger.	0.86	1.11	1	1
Lamiaceae	*Rydingia persica* (Burm.f.) Scheen &V.A.Albort	0.67	1.17	2	1
Lamiaceae	*Teucrium polium* L.	0.72	1.20	2	1
Lamiaceae	*Zataria multiflora* Boiss.	0.87	1.29	1	1
Lamiaceae	*Nepeta cataria* L.	0.89	1.51	1	1

Based on the independent samples *t*-test, women had more knowledge about the medicinal plants (*t* = 1.87, *p* = 0.04). Based on the field surveys, women in Kerman province are the preparers of the plant species for the medicinal applications, and it can be concluded that women had more practical experience in traditional medicine in this region. The findings of Sadat-Hosseini et al. in the southern part of this region also confirmed our results [10].

The results of ANOVA showed that there were significant differences between the three age groups (*F* = 3.17, *p* = 0.02), and different levels of education (*F* = 2.56, *p* = 0.03). Also, based on Duncan’s test, the two older age groups (older than 45 years old) with low level of education had more traditional knowledge about the medicinal plants. Based on these results and other reports like that of Hu et al., despite the importance of the traditional medicine for the older inhabitants, the younger generation does not show interest, which means that the ethnobotanical knowledge does not further flourish [2].

Finally, based on the results of ANOVA and Duncan’s tests, occupation of the informants had a significant effect on their traditional medicinal knowledge (*F* = 4.19, *p* = 0.01), and genuine information belonged to the herbal healers, nomadic people, and villagers, respectively. Field surveys revealed that herbal healers, due to their job requirement, record and learn the relevant knowledge of the other ethnic groups like nomadic and villagers and usually have more comprehensive traditional knowledge.

### Ethnopharmacological knowledge of tribes and different area

In the hot and dry regions of Kerman province like Kahnuj, Roodbar, Anbarabad, Qale Ganj, Manojan, Faryab, Bam, Fahraj, Narmashir, Rigan, and plain part of Jiroft, most inhabitants are from Baluch tribe and the natives of Jiroft and Kahnooj. Based on the results, medicinal plants such as *Berberis integerrima*, *R. persica*, *Calotropis procera*, and *R. stricta* are widely used to treat dermatological diseases. The rate of drug abuse in these regions is more than in mountainous areas because these cities are in the neighborhood of Afghanistan and the availability of drugs is thus higher. Hence, medicinal plants such as the plant from the genus *Achillea* (*A. wilhelmsii*, *A. eriophora*, *A. santolinoides*), *Berberis integerrima*, and *R. persica* are used individually or in combination by the locals for stopping drug abuse.

Tribal communities and folk people, who live in the mountainous areas such as Hezar, Sirch, and Jebal barez, mainly use the herbal medicine for the treatment of gastrointestinal disorders. Based on the field surveys and discussion with the herbal healers, the main food of the nomadic people is milk and its derivatives especially curd and buttermilk, and they do not have a diverse diet. There is a significant relationship between the consumption of low-diversity diets and the risk of non-communicable diseases [54, 55].

### New traditional medicinal uses

According to the in-depth comparison of the current ethnobotanical findings with previous national reports, a large volume of unrecorded traditional medicine knowledge was gathered. A major implication of the current study is identification of traditional medicinal use of 292 plants in the Kerman province and 201 plants species in the Persian ethnobotany for the first time. This unrecorded knowledge is summarized based on the plant families as follows:

Amaranthaceae (*Amaranthus retroflexus* for the treatment of jaundice; *Anabasis aphylla* and *Seidlitzia rosmarinus* as traditional washing powders), Apiaceae **(***Eryngium billardieri* and *Eryngium bungei* in pain relief, *Prangos ferulacea* as parasite repellents); Apocynaceae (*Cionura erecta* for sore throat and cough, *Rhazya stricta* for joint pains and body ache, *Calotropis procera* in the healing of skin disorders like eczema); Asteraceae (*Launaea acanthodes* as intestinal parasite repellents, *Artemisia* spp*.* for the treatment of gastric infection and stomachache, *Calendula officinalis* for the treatment of pterygium); Boraginaceae (*Cordia myxa* for common cold, sore throat and kidney stone); Ephedraceae (*Ephedra distachya* and *Ephedra foliata* for peptic ulcer and as materials in traditional tannery), Euphorbiaceae **(***Euphorbia serpens* for eczema); Fabaceae (*Astracantha lateritia* for hair tonic and eczema, *Prosopis cineraria* for eczema and in traditional tannery, *Prosopis farcta* in preventing nose bleeding) Lamiaceae (*Dracocephalum polychaetum* as potent and multipurpose medicinal plant); Plantaginaceae (*Plantago amplexicaulis*, *Plantago gentianoides* and *Plantago indica* for constipation and jaundice); Polygonaceae (*Pteropyrum aucheri* in healing of infectious wounds); Ranunculaceae (*Clematis ispahanica* in healing of eczema and psoriasis); Rosaceae (*Rosa moschata* as nerve tonic, *Prunus scoparia* in cancer prevention); Rubiaceae (*Plocama aucheri* in reducing rheumatic pain and blood sugar); Rutaceae (*Citrus limon* and *Citrus aurantium* for the treatment of eye diseases and making the traditional kohl); Salvadoraceae (*Salvadora oleoides* as parasite repellent). Tamaricaceae (*Tamarix aphylla* and *Tamarix kotschyi* in healing of eczema and skin disease); Thymelaeaceae (*Daphne oleoides* in traditional dyeing); Violaceae (*Viola odorata* for chronic cough and as expectorant); and Zygophyllaceae (*Zygophyllum eurypterum* and *Zygophyllum fabago* as lactiferous and vermicide). These findings highlight the importance of the documentation of such valuable ethnobotanical information. Also, some of these medicinal plants can be targeted for pharmacological and bioactive studies with the aim of identifying phytochemical content and therapeutic applications.

## Conclusion

Our extensive study in Kerman as the vastest province in Iran with 23 cities, 171,993 square kilometers area, and 89 tribal communities revealed rich traditional medicinal knowledge of its local populations. Traditionally, they used 402 medicinal plant species in 73 families to meet their pharmacological needs. Besides the common oral and topical utilization of the crude herbal drugs, dressing and bath with the medicinal plants are the exceptional mode of application in Kerman province. The highest ICF values belonged to diabetes, digestive, skin, and respiratory disorders, respectively.

Our findings suggested that Asteraceae and Apiaceae plants were dominantly used for the treatment of gastrointestinal disorders, Lamiaceae plants for respiratory and gastrointestinal ailments, and Apocynaceae plants for dermatological problems.

For several medicinal plants with high use reports such as *Cionura erecta*, *Tecomella undulata*, and *Launaea acanthodes*, scanty pharmacological and phytochemical data has been reported. On the other hand, the top list included *Rhazya stricta*: wound healing; *Calotropis procera*: eczema; *Berberis integerrima*: diabetes and addiction cessation; *Dracocephalum polychaetum*: stomachache, diarrhea, detoxification and strengthening body; *Rydingia persica*: *leaving* addiction; *Launaea acanthodes*: parasite repellent; *Cionura erecta*: expectorant; and *Tecomella undulate*: skin ailments, eczema, and diabetes. These results highlight the need for further bioactive and phytochemical studies on the mentioned medicinal plants. Finally, some frequently used medicinal plants like *Cionura erecta*, *Dracocephalum polychaetum*, and *Tecomella undulate* are endangered and restricted in small parts of their habitats. Therefore, urgent conservation measures are needed.

## Data Availability

All data generated or analyzed during this survey are included in this article.

## Abbreviation

*ITM*: Iranian traditional medicine
